# Cas9‐ and Cas12a‐mediated excision and replacement of the celiac disease‐related α‐gliadin immunogenic complex in hexaploid wheat

**DOI:** 10.1111/pbi.70200

**Published:** 2025-06-15

**Authors:** Miriam Marín‐Sanz, Susana Sánchez‐León, María H. Guzmán‐López, Colby G. Starker, Daniel F. Voytas, Francisco Barro

**Affiliations:** ^1^ Department of Plant Breeding Institute for Sustainable Agriculture (IAS‐CSIC) Córdoba Spain; ^2^ Department of Genetics, Cell Biology and Development, Center for Precision Plant Genomics University of Minnesota St. Paul MN USA

**Keywords:** 33‐mer, celiac disease, CRISPR, dsODN replacement, wheat, α‐gliadins

## Abstract

Celiac disease (CD) is a chronic enteropathy affecting approximately 1% of the global population. Wheat α‐gliadins are a major contributor to the autoimmune response, as they contain one of the most immunogenic peptides, the 33‐mer, along with numerous variants. In this study, we used CRISPR/Cas technology to mutate genes encoding α‐gliadins. This approach employed paired sgRNAs to precisely excise immunogenic regions while preserving non‐immunogenic sequences within the α‐gliadins. Furthermore, we replaced the excised region with an α‐gliadin‐based double‐stranded oligodeoxynucleotide (dsODN) designed with nucleotide changes to reduce immunoreactivity and increase peptidase cleavage sites. Two different CRISPR systems, Cas9 and Cas12a, were applied to generate wheat protoplasts and plants with non‐immunogenic regions. Cas9 demonstrated superior performance in terms of editing frequency, excision and replacement of immunogenic fragments. However, the Cas12a nuclease (Cpf1) showed promising editing efficiency, offering the potential for future wheat editing applications. Using the Cas9 system, we achieved a 74.2% excision rate of the 33‐mer in wheat plants. Subsequent analyses showed a significant reduction in the reactivity to the G12 monoclonal antibody, capable of identifying the 33‐mer peptide and a decrease in the prolamin levels compared to the wild‐type. Additionally, we developed a high‐throughput sequencing‐based software specifically designed to identify mutations in multi‐copy gene families. This innovative tool enabled fast, parallel screening of the samples in this study and facilitated the identification of the specific editing patterns produced by the designed constructs.

## Introduction

Over the past few decades, genes encoding for gluten proteins have emerged as an attractive target for genetic engineering to develop wheat varieties for individuals with celiac disease (CD) and other wheat‐related disorders (WRDs). The CD is the best known among a group of pathologies triggered by the ingestion of foods containing gluten from wheat, barley or rye. Overall, WRDs could affect between 6 and 12% of the population in Western countries (Singh *et al*., 2018). In CD, the immune response is produced by the partial degradation of gluten proteins, which produce peptides recognized by antigen‐presenting cells (APCs) and elicit an immune response by CD4+ T cells (Ludvigsson *et al*., 2013; Sollid *et al*., 2020). As a consequence of the great heterogeneity of gluten proteins, there are many epitopes recognized by T cells, but all of them have in common a central core of 9 amino acids which interacts with pockets within the peptide‐binding groove of the human leukocyte antigen DQ (HLA‐DQ) molecules. The HLA‐DQ are major histocompatibility complex class II (MHC‐II) molecules that play a crucial role in the immune response to gluten in CD. These molecules are expressed on the surface of APCs, which are responsible for peptide loading and presenting gluten‐derived peptides to CD4+ T cells in the intestinal mucosa (Fallang *et al*., 2009; Jabri *et al*., 2014). Gluten proteins consist of monomeric and polymeric families known as gliadins and glutenins, respectively. Gliadins are further divided into three structural groups by their mobility in acid polyacrylamide gel electrophoresis (A‐PAGE): ω‐, α/β‐ and γ‐gliadins (Shewry *et al*., 2009). DQ2‐restricted T‐cell epitopes are gluten‐derived peptide sequences that specifically bind to the HLA‐DQ2.5 molecules present on APCs, initiating the immune response in individuals with CD, and are distributed in regions mainly in the gliadin proteins with high content of proline and glutamine residues (Arentz‐Hansen *et al*., 2002; Sollid *et al*., 2020). The α‐gliadins contain a region rich in DQ2.5‐restricted epitopes responsible for almost 90% of the immunogenic response in CD after eating foods containing wheat (Tye‐Din *et al*., 2010). This region is known as 33‐mer and comprises six overlapping copies of three DQ2.5 epitopes (PFPQPQLPY, DQ2.5_glia_α1a, one copy; PYPQPQLPY, DQ2.5_glia_α1b, two copies; and PQPQLPYPQ, DQ2.5_glia_α2, three copies), along with an additional downstream epitope (FRPQQPYPQ, DQ2.5_glia_α3). The 33‐mer is highly resistant to digestion, favouring the recognition of these epitopes by the HLA‐DQ molecules in the intestine (Ozuna *et al*., 2015; Shan *et al*., 2002; Sollid *et al*., 2020). This is particularly relevant for the DQ2.5_glia_α2 and DQ2.5_glia_α1a epitopes, which are central in CD pathogenesis, as they are recognized by all CD patients and are considered immunodominant (Tye‐Din *et al*., 2010). The high immunogenicity of α‐gliadins is also explained by the presence of the p31‐43 peptide, responsible for eliciting the CD innate immune response (Maiuri *et al*., 2003).

The α‐gliadins are encoded by a gene family with multiple copies across the hexaploid wheat genome, most of them arranged in tandem. The α‐gliadins from the D‐genome are the most immunogenic and the only ones that contain the full 33‐mer peptide (Marín‐Sanz *et al*., 2023; Molberg *et al*., 2005; Ozuna *et al*., 2015) while those from the B‐genome contain no or few CD epitopes and are less immunogenic (Marín‐Sanz *et al*., 2023; van Herpen *et al*., 2006). Regarding the α‐gliadins from the A‐subgenome, it contains many genes presenting the p31‐43 coding region, genes with 0–2 DQ epitopes and genes with a high prevalence of premature stop codons (Marín‐Sanz *et al*., 2023). Although a small number of the α‐gliadin genes harbour the six overlapping 33‐mer epitopes, many of them also expand highly immunogenic variants containing between one and five CD epitopes (Marín‐Sanz *et al*., 2023).

It is, therefore, not surprising that the α‐gliadins represent an appealing target for reducing wheat immunogenicity, and various microbial, enzymatic, pharmaceutical, plant breeding, molecular biology and biotechnology approaches have been carried out (reviewed in Rosell *et al*., 2014; Sharma *et al*., 2020; Verma *et al*., 2021). Gene silencing by RNA interference (RNAi) was used for successful down‐regulation of α‐, γ‐ and ω‐gliadins, resulting in a remarkable 98.1% reduction in total gluten content (Gil‐Humanes *et al*., 2010). These lines were tested in non‐celiac wheat sensitivity (NCWS) patients (Haro *et al*., 2018), and in DQ2.5 CD patients, demonstrating no immunological response upon consuming bread made from these RNAi lines (Guzmán‐López *et al*., 2021b). A non‐transgenic approach for lowering α‐gliadin epitopes was reported using CRISPR/Cas for introducing targeted mutations in the α‐gliadin genes using constructs with one single guide RNA (sgRNA), leading to the mutation of 35 out of 45 genes (Sánchez‐León *et al*., 2018). In that strategy, the cleavage sites were 5′ of the 33‐mer coding region, where a frameshift‐inducing mutation would disrupt the correct translation of α‐gliadin genes and therefore prevent protein deposition in the grain. Although this loss of functionality is entirely desirable to eliminate gluten immunogenicity, it could represent a loss of function and a decrease in the technological properties of wheat. However, a much more desirable situation is to specifically remove the immunogenic region of the α‐gliadins, and even replace or rewrite this region with another sequence of similar characteristics but not immunogenic.

In CRISPR/Cas systems, an RNA‐guided Cas nuclease induces a double‐strand break (DSB) 5′ of the protospacer adjacent motif (PAM), which can be repaired through either the non‐homologous end joining (NHEJ) pathway or homology‐directed repair (HDR). While NHEJ induces insertions/deletions (InDels) that may lead to frameshift mutations in the target gene, HDR uses a template to introduce gene knock‐ins or sequence replacements (Puchta and Fauser, 2014). Furthermore, the use of paired sgRNAs would allow the excision of specific fragments and/or their replacement if the appropriate templates are provided. The excision of DNA fragments between paired sgRNA binding sites in wheat was previously reported by Cui *et al*. (2019), obtaining deletions of 95 bp. In rice and Arabidopsis, large deletions were reported by using two nuclease‐targeted sites provoking large chromosomal excisions and loss‐of‐function alleles, respectively (Pauwels *et al*., 2018; Zhou *et al*., 2014). In fact, we have recently obtained large excisions from sgRNA‐to‐sgRNA when a multiplexed Cas9 system was employed to edit ω‐ and γ‐gliadin genes (Sánchez‐León *et al*., 2024), paving the way to design sgRNA flanking the highly immunogenic regions of the α‐gliadin genes.

Oligodeoxynucleotide (ODN)‐assisted genome editing exploits these repair pathways to introduce long sequences and gene substitutions. Particularly, when employing a double‐stranded ODN (dsODN), it is embedded in the DSB sites through the NHEJ pathway (Tsai *et al*., 2015). The integration of dsODN via NHEJ can occur through two distinct repair pathways: canonical NHEJ (c‐NHEJ) and microhomology‐mediated end joining (MMEJ). c‐NHEJ directly ligates the dsODN at the break, while MMEJ utilizes short microhomologous sequences flanking the break (Bhargava *et al*., 2016; Yao *et al*., 2017). Notably, NHEJ‐mediated modifications exhibit higher efficiency compared to HDR modifications (Chen *et al*., 2019), making them favourable for targeted insertions. Despite limited research on targeted insertions in animal and plant systems, recent studies have explored NHEJ‐mediated dsODN insertions, particularly in plants (Kumar *et al*., 2023; Lu *et al*., 2020). In the present study, we employed paired sgRNAs flanking the 33‐mer coding region to excise and replace it with non‐immunogenic fragments using dsODNs as template sequences.

For the excision of the immunogenic regions and replacement, both Cas9 and Cas12a systems were tested. The Cas12a is a class 2‐type V CRISPR/Cas system (*Lachnospiraceae bacterium* Cas12a, LbCas12a) that has been employed for plant genome editing (Bandyopadhyay *et al*., 2020). Unlike other systems, Cas12a recognizes a T‐rich PAM (5′‐TTTN‐3′) at the 5′ end of the target DNA (Zetsche *et al*., 2015). This feature significantly broadens the spectrum of editing sites in the genome, complementing those recognized by Cas9 from *Streptococcus pyogenes* (SpCas9), which typically targets NGG‐PAM sequences. Other features, such as the multiplexing ability, the relative position of cleavage sites with respect to the PAM and the optimal temperature for the activity of LbCas12a versus SpCas9, make each of them more suited for use in some species or experimental designs (Malzahn *et al*., 2019; Wang *et al*., 2017). The Cas12a system was previously used for plant genome editing in rice, Arabidopsis and wheat, showing that this system is a powerful genome editing tool for polyploid plants in the last case (Kim *et al*., 2021; Tang *et al*., 2017; Wang *et al*., 2017).

In this study, we have used Cas9 and Cas12a to remove and replace the 33‐mer coding region with dsODNs in wheat protoplasts. In addition, the 33‐mer peptide and its variants were also successfully removed in wheat plants. To address the challenge of identifying CRISPR mutations within the α‐gliadins gene family, we developed a novel automated pipeline based on reference gene family alignment to detect sgRNA‐specific edits, as well as large deletions covering multi‐sgRNA binding sites in different sample types. This pipeline leveraged amplicon sequencing and was implemented in a high‐performance computing (HPC) cluster for parallel processing.

## Results

### Design of Cas9 and Cas12a edits in α‐gliadin genes

**Figure 1:**
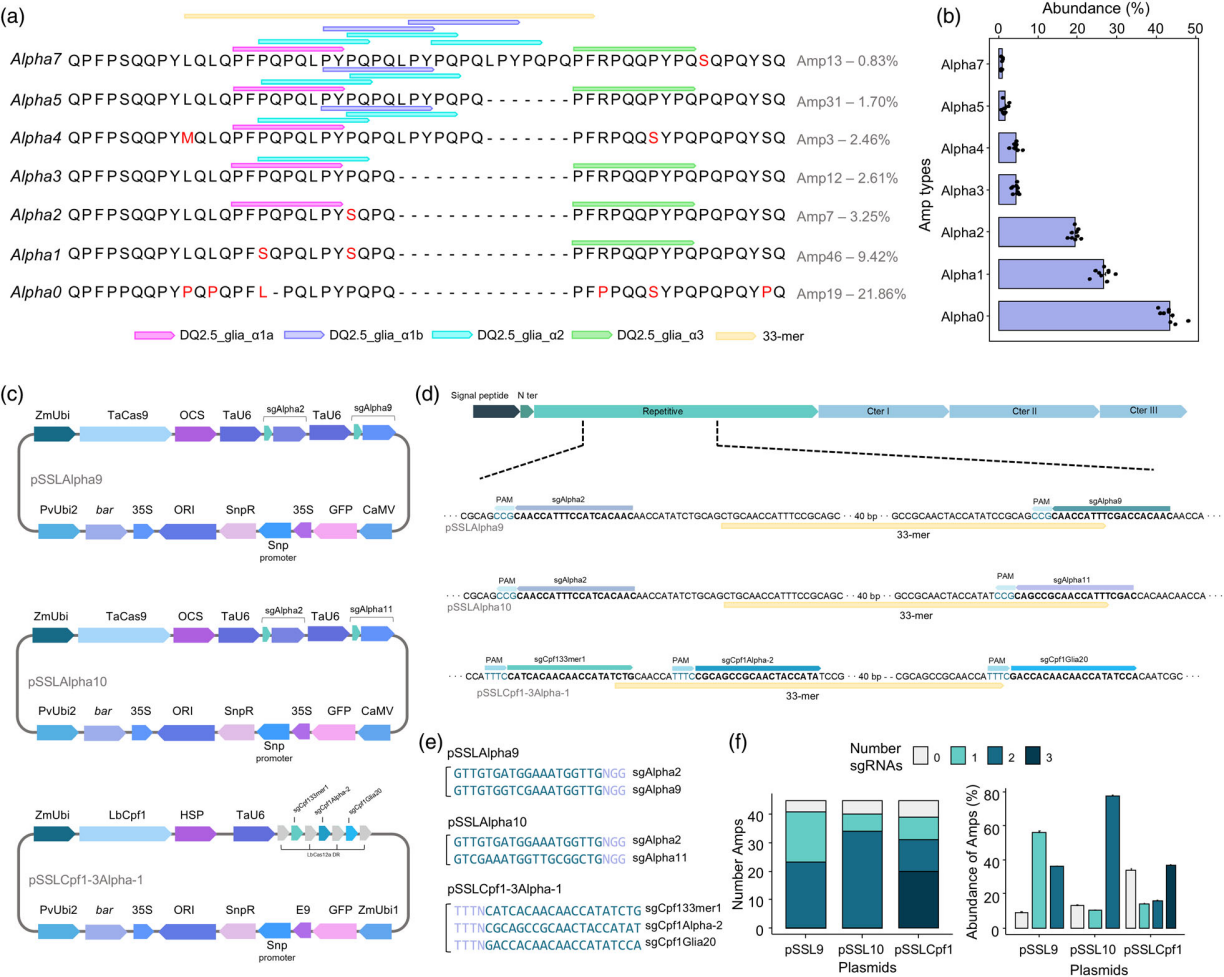
Types of α‐gliadin genes and plasmids and sgRNAs design. (a) Alignment of Amp types of α‐gliadin protein sequences. The most abundant reference Amp per Alpha type was selected for the representation, and its ID and abundance are indicated on the right side. Pseudogenes were not included in the representation. The DQ epitopes and the 33‐mer peptide positions are shown upper the sequences. The mismatches are indicated in red. (b) Abundance (%) of each Amp type of α‐gliadins in the wild‐type (BW208). The mean and standard error of nine biological replicates are represented, as well as the values for each replicate. (c) Scheme of the plasmids used for protoplasts and plant transformation. (d) Position of the sgRNAs per plasmid in the α‐gliadin sequence with the complete 33‐mer. (e) sgRNA sequences for each plasmid. (f) Number of reference Amps of α‐gliadins with 0–3 hits of different sgRNAs per plasmid and their mean abundance in BW208. The bar in the right plot indicates the standard error. pSSL9: pSSLAlpha9, pSSL10: pSSLAlpha10, pSSLCpf1: pSSLCpf1‐3Alpha‐1.

To analyse the CRISPR edits in protoplasts and plants, we used a database comprising 45 α‐gliadin reference amplicons (Amps) from BW208 (Sánchez‐León *et al*., 2021). These Amps represent unique sequences repeated throughout the genome at varying lengths and frequencies, reflecting their abundance. For downstream analysis, the reference Amps were categorized into different types based on the number of CD‐immunogenic epitopes they contain, as described by Sollid *et al*. (2020). As shown in Figure 1a, the Alpha0 type lacked CD epitopes, while Alpha7 contained the complete 33‐mer. We calculated the abundance of each Amp type using nine biological replicates (Guzmán‐López *et al*., 2021a; raw reads available in the BioProject NCBI database under accessions PRJNA354904 and PRJNA782791). As shown in Figure 1b, Alpha0 was the most abundant in BW208, followed by Alpha1 and Alpha2, whereas Alpha7 showed low abundance. These findings align with the abundance of Amp types reported in other bread wheat genotypes (Marín‐Sanz *et al*., 2023).

The Amp sequences were used to design the sgRNAs flanking the α‐gliadin immunogenic region to remove the 33‐mer peptide and replace it with non‐immunogenic sequences (Figure 1c–e). Designed sgRNAs were incorporated into three plasmids (Figure 1c): two for Cas9 and one for Cas12a. For Cas9, three guide RNAs were designed and combined into the two plasmids, with one sgRNA present in both. For Cas12a, the three guide RNAs target the entire 33‐mer region, with 2–3 sgRNAs mapping across the entire region depending on the α‐gliadin gene (Figure 1d).

Notably, the sgRNA targets on α‐gliadin genes varied across the three constructs assayed. For pSSLAlpha10, both sgRNA binding sites were present in numerous reference Amps, which are also highly abundant in the genome (Figure 1f), and only a few Amps showed no hits for these sgRNAs. Conversely, for pSSLAlpha9, a comparable number of reference Amps contained either both sgRNA binding sites or just one, with a smaller proportion of Amps lacking sgRNA binding sites altogether. Interestingly, Amps with only one sgRNA binding site were more prevalent (Figure 1f). Additionally, more than half of the Amps harboured all three sgRNA binding sites of pSSLCpf1‐3Alpha‐1, showing a similar level of abundance to those with any sgRNA binding site (Figure 1f).

### Editing efficiency and InDel patterns in wheat protoplasts

**Figure 2:**
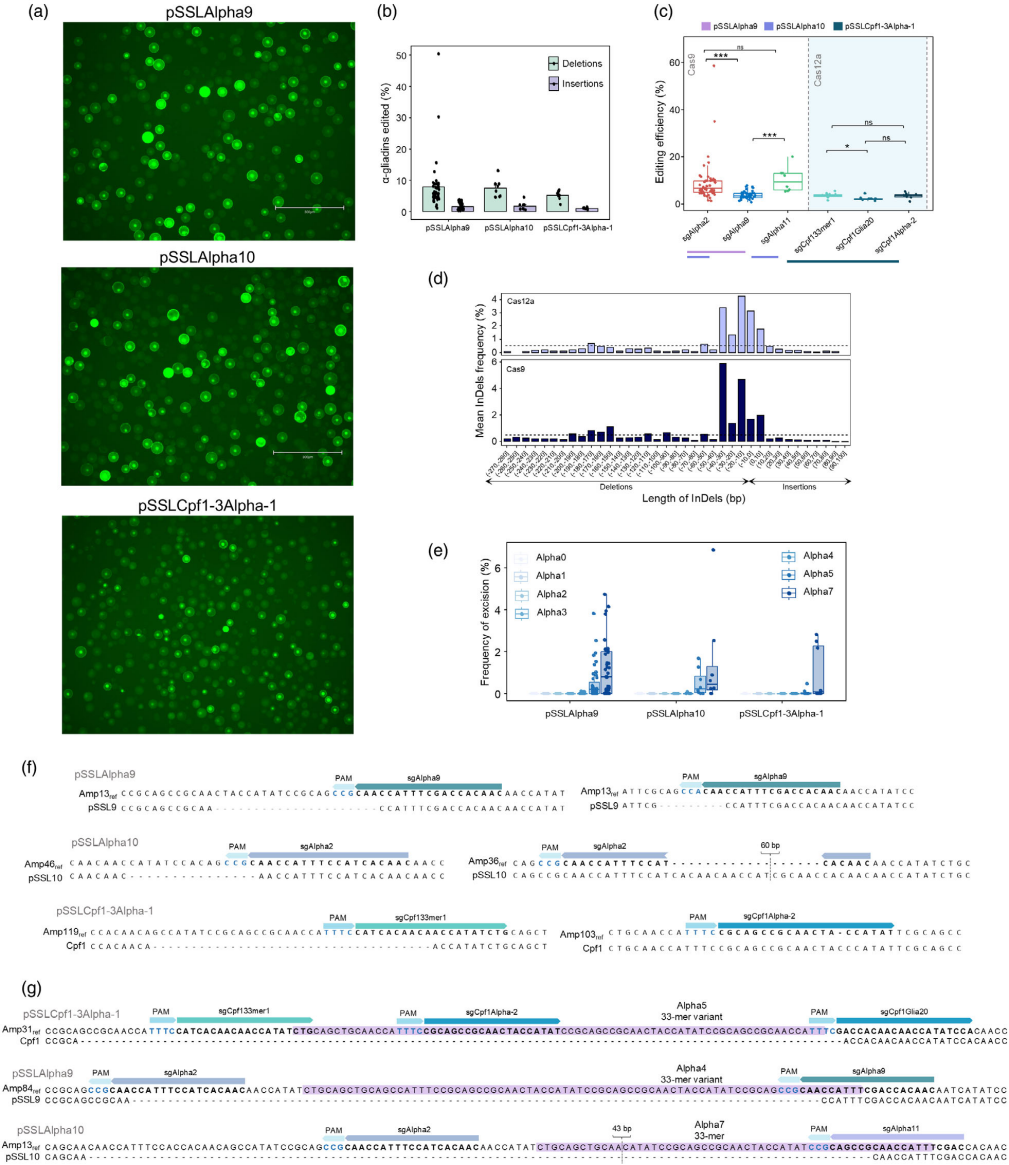
The α‐gliadin edits in wheat protoplasts. (a) Example of GFP fluorescence in transformed protoplasts with pSSLAlpha9, pSSLAlpha10 and pSSLCpf1‐3Alpha‐1. (b) Frequency of editing of the α‐gliadins (%) in protoplasts transformed with pSSLAlpha9, pSSLAlpha10 and pSSLCpf1‐3Alpha‐1 plasmids. The mean is represented, the bars represent the standard error, and the dots indicate the sample values. There were no significant differences in edition efficiency for deletions/insertions among plasmids used. (c) Edition efficiency of each sgRNA in protoplasts. ***P < 0.001; *P < 0.05; ns: not significant. (d) Mean of the frequency of each InDel by its length (bp) per nuclease in all the protoplast isolates. The frequency was calculated as the abundance of each InDel divided by the sum of the total InDels frequency per sample. A threshold of 0.5% was set to facilitate the interpretation of the figure. (e) Frequency of excision of the complete 33‐mer (in the Alpha7 Amp type) and the 33‐mer peptide variants (in Alpha1‐5 Amp types) in protoplasts. (f) Examples of InDels in protoplast DNA provoked by the CRISPR editing system. (g) Examples of excision of the 33‐mer peptide variants in protoplasts transformed with pSSLAlpha9 (pSSL9), pSSLAlpha10 (pSSL10) and pSSLCpf1‐3Alpha‐1 (Cpf1).

A total of 59 preparations of leaf tissue‐derived protoplasts were completed and subjected to PEG‐mediated transfection with pSSLAlpha9, pSSLAlpha10 and pSSLCpf1‐3Alpha‐1 constructs (Table S1). The average transformation efficiency of protoplasts was approximately 57% (Figure 2a), consistent with previous wheat studies (Zhong *et al*., 2023). Protoplast DNA samples underwent amplicon sequencing covering the region of the α‐gliadin genes targeted by the sgRNAs. To identify the InDels, we developed the ampAnalysis software (https://github.com/MiriamMarinS/ampAnalysis). This software provides efficiency metrics and other statistics related to the paired sgRNA CRISPR system for the wheat α‐gliadin family gene members, facilitating the identification of InDels through local and global alignments against a variety‐specific reference sequences database (Figure S1). The InDels identification in gliadins' gene families has been previously faced by different protocols, including the use of droplet digital PCR (ddPCR) to detect both large and small mutations (Jouanin *et al*., 2020), as well as in‐solution gluten exome capture (Jouanin *et al*., 2019). Due to next‐generation sequencing (NGS)‐mediated analyses enabling the qualitative and quantitative identification of small, medium and large InDels, including larger excisions, amplicon sequencing of a gene family is an efficient alternative to pools of edited plant cells. Several tools have been developed for the identification of CRISPR mutations using amplicon sequencing, including CRISPResso2 (Clement *et al*., 2019), CRISPR‐GA (CRISPR‐Genome Analyzer), Cas‐Analyzer (Güell *et al*., 2014), CRISPRpic (Lee *et al*., 2020) and CrispRVariant (Lindsay *et al*., 2016). However, accounting for polymorphisms between wheat genotypes and the sequence variability within a gene family, the use of multi‐reference wild‐type‐exclusive databases is needed. Therefore, a bioinformatics pipeline based on amplicon sequencing was reported for characterizing gene family InDels in wheat lines (Guzmán‐López *et al*., 2021a). This protocol effectively identified the different mutation profiles of the CRISPR lines obtained with one sgRNA targeting α‐gliadin genes (Sánchez‐León *et al*., 2018). Nonetheless, human intervention was still required during the InDels identification process, which would slow down mutation analysis. The software presented in the present work was designed to overcome the limitations exposed above. Overall, 74.5% of reads were aligned to the BW208 Amps database using local alignment, with an additional 2.1% mapped through global alignment within protoplasts samples. The last one was more effective for identifying large deletions. In addition to InDels found in sgRNAs with perfect match in the α‐gliadin genes, editions can be found in sgRNAs' binding sites with up to three mismatches (Anderson *et al*., 2015; Cui *et al*., 2019). For that reason, sgRNAs were designed to avoid off‐targets including the ones with mismatches when using the bread wheat reference genome (Appels *et al*., 2018), and on‐target InDels were searched in non‐perfect sgRNA binding sites.

Notably, deletions were observed at the highest frequency across experiments, regardless of the plasmid used for transformation (Figure 2b). Within each plasmid, we observed varying ranges of editing frequencies, with no significant differences between InDels when comparing the constructs. Protoplasts transformed with pSSLAlpha9 exhibited editing efficiencies ranging from 1.4% to 50.9% (Table 1, Figure 2b). Protoplasts transformed with pSSLAlpha10 reached an editing frequency of 17.2%, while those transformed with the Cas12a nuclease (pSSLCpf1‐3Alpha‐1 plasmid) achieved a maximum editing frequency of 7.7% (Table 1). These results clearly showed a considerable variation in editing frequency within the protoplasts transformed with each plasmid, particularly notable for pSSLAlpha9. This variability may be attributed to batch effects during the protoplast isolation, as previously reported by Cui *et al*. (2019).

**Table 1:** Range of variation of the percentage of α‐gliadin gene family editing in protoplasts and plants per plasmid

Nuclease	Plasmid	Editing of α‐gliadins (%)	α‐gliadins with deletions (%)	α‐gliadins with insertions (%)^a^
Protoplasts				
Cas9	pSSLAlpha9	1.39–50.94	1.14–50.38	0.31–3.86
Cas9	pSSLAlpha10	5.27–17.16	4.7–13.15	0.54–4.63
Cas12a	pSSLCpf1‐3Alpha‐1	4.96–7.69	4.32–6.7	0.99–1.17
Plants				
Cas9	pSSLAlpha9	3.91–34.24	3.9–34.21	0.003–5.06
Cas9	pSSLAlpha10	5.33–7.07	5.4–6.73	0.23–0.42
Cas12a	pSSLCpf1‐3Alpha‐1	3.14–3.82	3.13–3.81	0.01–0.03

a The insertions comprise targeted and non‐targeted ones.

When comparing the editing efficiency of individual sgRNAs, sgAlpha2 exhibited significantly higher editing efficiency than sgAlpha9, whereas sgAlpha11 had comparable efficiency to sgAlpha2 (Figure 2c). Overall, the three sgRNAs of pSSLCpf1‐3Alpha‐1 exhibited lower editing efficiencies compared to that of Cas9. However, sgCpf133mer demonstrated significantly higher efficiency than sgCpf1Glia20 (Figure 2c).

The most frequent InDels found in single sgRNAs were short/medium‐sized, ranging from ‐40 to 10 bp, regardless of the construct and nuclease used (Figure 2d,f). Many editing events included deletions covering the binding sites of two or three sgRNAs (Figure 2d,g). Although deletions encompassing the excision of the immunogenic fragments were less frequent than the short/medium‐sized deletions affecting only one sgRNA, we succeeded in removing the 33‐mer coding region as well as shorter 33‐mer variant fragments, present in other α‐gliadin genes (Figure 2e,g). The frequency of excision of all 33‐mer variants was calculated based on the total abundance of each Amp type. Notably, the complete 33‐mer (Alpha7) exhibited a higher excision frequency compared to its variants in protoplasts transformed with any of the constructs, followed by the variant containing five CD epitopes (Alpha5) for the Cas9‐containing constructs (Figure 2e).

### Replacement of 33‐mer variants with dsODN in wheat protoplasts

Six dsODN sequences were designed to replace the 33‐mer peptide with shorter, less immunoreactive versions (Figure S2a). Moreover, these dsODNs incorporated selective single nucleotide polymorphisms (SNPs) to abolish any possible immunoreactive properties. Previous *in vitro* studies with peripheral blood mononuclear cells (PBMCs) demonstrated that substituting specific amino acids in CD epitopes significantly reduced the stimulatory response compared to the canonical epitope (Ruiz‐Carnicer *et al*., 2019). Moreover, all three DQ2.5 epitopes share the ‘core’ PELP amino acid sequence, where lysine substituted for any position reduced mean bioactivity to <5% (Anderson *et al*., 2006). It is well known that the 33‐mer region is highly resistant to gastric enzymes. Additionally, modifications were introduced in the dsODNs to increase the cleavage sites for gastric enzymes, enhancing the breakdown of the target region. Comparing the peptide encoded by the dsODNs with the complete 33‐mer region, a greater number of cleavage sites in the dsODN‐encoded peptides can be found (Figure S3).

Two lengths of dsODN, 51 and 75 base pairs (bp), were designed considering Cas9 and Cas12a cleavage outcomes (Figure S2a). These lengths aimed to generate smaller protein products after replacement while maintaining in‐frame α‐gliadin proteins. Two Cas9 dsODN sequence end variants, one bp overhang (OV) and blunt end (BE), were also tested for potential differences in replacement efficiency (Figure S2a,b).

**Figure 3:**
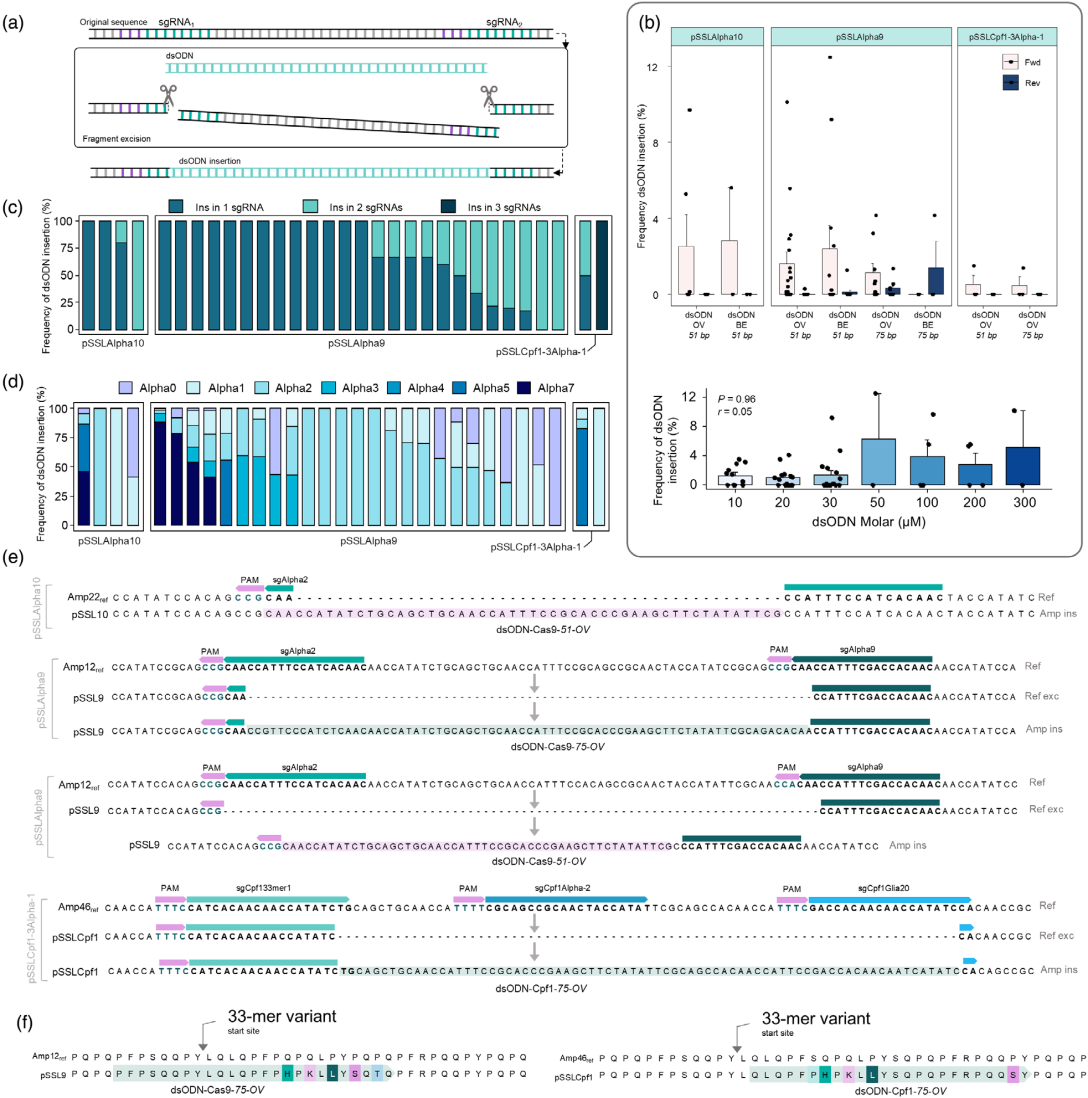
The highly immunogenic α‐gliadin region replacement by dsODN targeted insertion. (a) Scheme of the expected dsODN‐Cas insertion procedure. (b) Frequency of forward (Fwd) and reverse (Rev) insertion of dsODN in protoplasts transformed with pSSLAlpha9, pSSLAlpha10 and pSSLCpf1‐3Alpha‐1. The mean is represented, the bars indicate the standard error, and the dots represent each sample value. Frequency of dsODN insertion (%) per concentration level (μM). The Kruskal–Wallis non‐parametric test was performed to analyse the differences among concentration levels and the Kendall correlation coefficient was indicated (*r*). (c) Frequency of dsODN insertion per number of sgRNAs covered. (d) Frequency of insertion of dsODN per Amp type. (e) Examples of dsODN insertion in protoplasts transformed with pSSLAlpha9, pSSLAlpha10 and pSSLCpf1‐3Alpha‐1. (f) Examples of final protein product after dsODN insertion. The mismatches in the protoplasts Amps against the references are highlighted. Amp ins, Amp with the dsODN insertion; BE, blunt end; dsODN, double‐stranded oligodeoxyribonucleotide; OV, overhang; Ref exc, reference with the prior sequence excision; Ref, reference sequence. The frequency of insertion of dsODN was calculated per percentage of total insertions (targeted and non‐targeted) in each sample.

The replacement of the 33‐mer and its variants requires the presence of flanking sgRNAs, limiting targets among α‐gliadin types (Figure 3a, Figure S2c). The Alpha0 type is particularly receptive to dsODN insertion when the plasmid pSSLAlpha10 is used, while Alpha1 and Alpha2 are not. However, types Alpha3, Alpha4, Alpha5 and Alpha7, containing more epitopes, showed potential for dsODN substitution across all plasmids. Particularly interesting is the Alpha4 type, which has more sgRNA sites flanking the four epitopes coding region in the pSSLCpf1‐3Alpha‐1 construct (Figure S2c).

Successful targeted replacement of the designed fragment was achieved in multiple instances, resulting in perfect, in‐frame substitutions. However, the dsODN insertion frequency varied between and within experiments (Figure 3b). Most targeted insertions occurred in the forward sense across all constructs, except for the dsODN‐Cas9‐75‐BE in pSSLAlpha9 experiments (Figure 3b). Interestingly, no significant differences in insertion frequencies were observed between dsODN lengths or OV/BE dsODN types (Figure 3b). Although the dsODNs were used at different molarities (10–300 μM), no correlation was found between the targeted insertion frequency and molarity (*r* = 0.05) (Figure 3b).

Two scenarios for dsODN insertions were identified: (i) 5′ of the PAM site of one sgRNA binding site or (ii) between two sgRNA binding sites after removing the sequence between both sites (Figure S1). Both scenarios were observed in protoplasts transformed with all three plasmids. In pSSLAlpha9 and pSSLAlpha10 transformations, targeted insertions predominantly occurred within one sgRNA binding site (Figure 3c). In one pSSLCpf1‐3Alpha‐1 experiment, all dsODN insertions encompassed the three sgRNAs of the construct (Figure 3c). An illustrative example of the first type of insertion was identified in the sgAlpha2 of protoplasts transformed with pSSLAlpha10 (Figure 3e). The second type, replacing the 33‐mer variants, was observed in protoplasts transformed with pSSLAlpha9 and pSSLCpf1‐3Alpha‐1 (Figure 3e). The resulting sequences were shorter than the original due to the length of the dsODNs compared to the excised fragment (Figure 3e). Protoplasts transformed with pSSLAlpha9 and pSSLAlpha10 predominantly showed substitutions of the immunogenic region harbouring one (Alpha1), two (Alpha2) and the complete 33‐mer (Alpha7) containing six CD epitopes. In contrast, pSSLCpf1‐3Alpha‐1 protoplasts did not have any Alpha7 substitution, but exhibited a high proportion of Alpha5 replacements (Figure 3d). The highly immunogenic region replacements obtained in this work resulted in protein sequences like one original α‐gliadin type, with only a few amino acid changes at specific strategic sites (Figure 3f).

### CRISPR/Cas‐mediated excision of 33‐mer in plants

To excise and replace the immunogenic region in wheat plants, immature embryos of bread wheat were transformed with the same constructs used for protoplasts and the dsODNs. All regenerated plants were screened by PCR and A‐PAGE gels for Cas9/Cas12a and possible alterations in the gliadin protein pattern. A total of 40, 9 and 173 T0 plants containing plasmids pSSLAlpha9, pSSLAlpha10 and pSSLCpf1‐3Alpha‐1, respectively, were obtained (Table 2). The A‐PAGE gliadins profile showed a reduction in either partial or nearly complete α‐gliadins in Cas9 plants transformed with pSSLAlpha9 and pSSLAlpha10 plasmids (Figure S4), particularly evident in the Alpha‐30 offspring. Conversely, plants transformed with pSSLCpf1‐3Alpha‐1 exhibited the absence of only a few bands within the α‐gliadins region (Figure S5).

**Table 2:** Number of lines generated in T0 and T1, and number of Cas9/Cas12a‐positive lines after PCR screening

Plasmid	T0 lines		T1 lines	
	All lines	Cas9/Cas12a +	All lines	Cas9/Cas12a +
pSSLAlpha9	113	40	46	21
pSSLAlpha10	33	9	6	4
pSSLCpf1‐3Alpha‐1	360	173	74	40

**Figure 4:**
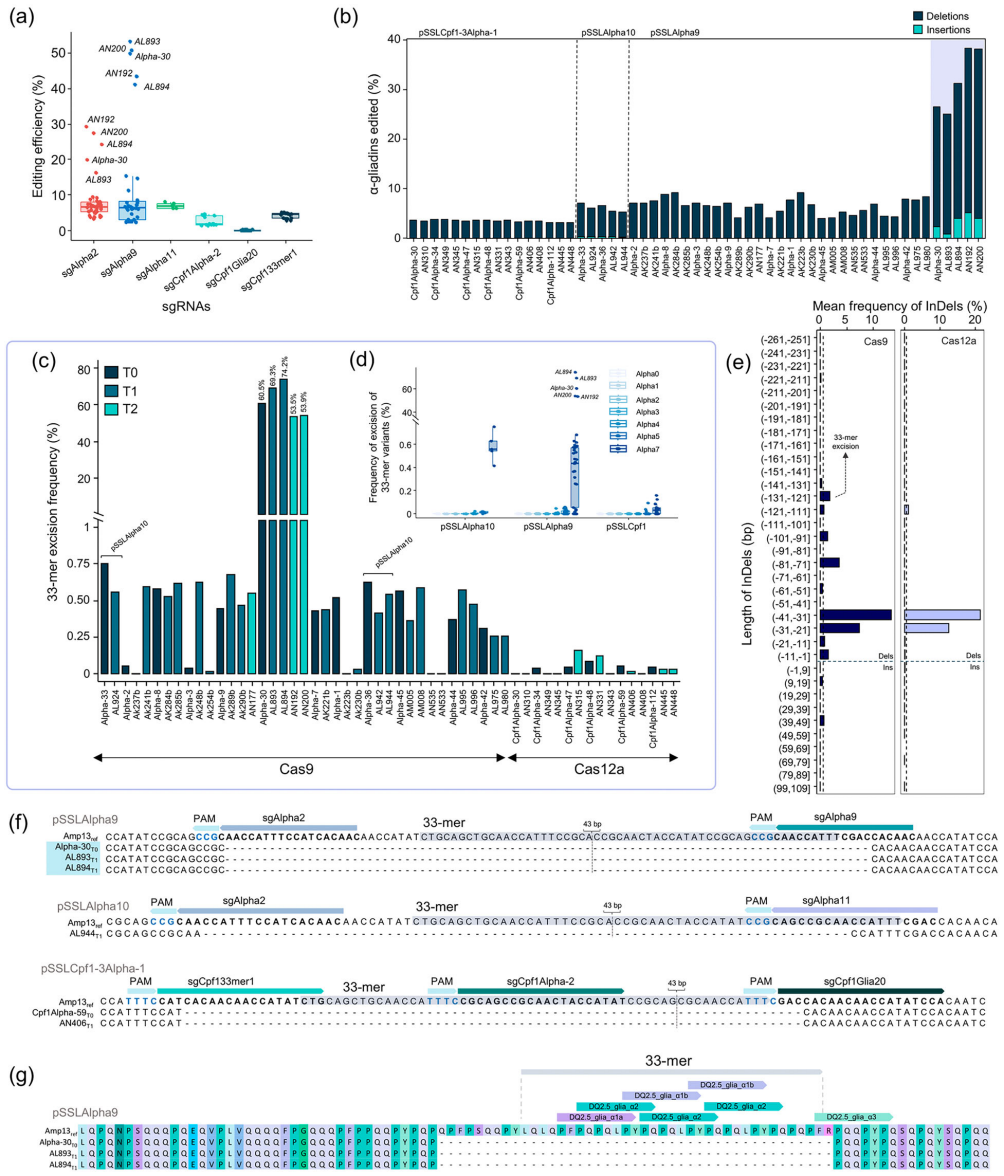
The 33‐mer region excision in wheat plants. (a) sgRNAs efficiency in terms of % of InDels in Amps targeted by each sgRNA in plants. The top 5 outliers for pSSLAlpha9 sgRNAs are indicated. (b) Percentage of the total α‐gliadins with insertions/deletions per plant. The Alpha‐30 and its offspring are highlighted in a purple box. (c) The frequency (%) of excision of the complete 33‐mer per plant. The lines not labelled as pSSLAlpha10 were transformed with pSSLAlpha9. (d) The frequency (%) of extraction of each 33‐mer variant per plant. The five labelled points corresponding to the Alpha‐30 and its offspring were not used to represent the boxplots. (e) Mean of the frequency of InDels per length in Cas9 and Cas12a plants. Dels, deletions; Ins, insertions. The frequency was calculated as the abundance of each InDel divided by the sum of the total InDels frequency per sample. A threshold of 0.5% was set to facilitate the interpretation of the figure. (f) Example of 33‐mer excision in Alpha‐30 and its offspring. (g) Representation of part of the putative α‐gliadin proteins with the 33‐mer extraction in Alpha‐30 and its offspring. The CD epitopes on the reference protein were represented.

In total, 55 lines comprising T0, T1 and T2 generations were subjected to amplicon sequencing of the α‐gliadins. Although the efficiency of sgRNAs varied widely among plants, many lines exhibited similar sgRNA efficiencies to those observed in protoplasts (Figure 4a). Notably, the plant named Alpha‐30—transformed with pSSLAlpha9—and its progeny displayed a marked abundance of InDels for sgAlpha2 and sgAlpha9 (Figure 4a). These lines showed the highest percentage of edited α‐gliadins, while those with pSSLCpf1‐3Alpha‐1 and pSSLAlpha10 exhibited lower levels of editing (Figure 4b, Table 1).

The most predominant deletion had a length of −36 bp in both Cas9 and Cas12a plants (Figure 4e). However, a broader range of InDel lengths was observed in Cas9 plants compared to Cas12a, with the interval between −141 to +49 bp showing the highest frequency of mutations (Figure 4e). Notably, a peak of large deletions of −129 bp corresponded to the complete 33‐mer excision caused by the deletion from sgAlpha2 to sgAlpha9 in Alpha‐30 and its progeny (Figure 4e). The 33‐mer fragment excision frequency ranged from 53.5% to 74.2% in these lines, whereas Cas12a plants showed frequencies below 1% (Figure 4c).

The removal of other 33‐mer variants was also achieved in plants, especially the one containing 5 epitopes (Alpha5). However, the frequencies of these variant removals were not notably high (Figure 4d). The highly prevalent ‐129 bp deletion in the Alpha‐30 line and its progeny was in‐frame, suggesting potential translation into functional proteins without the 33‐mer immunogenic peptide. The putative α‐gliadin protein of these CRISPR lines is depicted in Figure 4g. Conversely, the low‐frequency 33‐mer removal found in line AL944 (pSSLAlpha10) did not result in an in‐frame deletion but retained the last bases of this region (Figure 4f).

Despite co‐transformations with the Cas‐containing plasmids and the dsODNs used in the protoplast experiments, sequencing results did not identify plants carrying the insertion of dsODNs in replacement of the 33‐mer sequences or their variants.

### CD epitopes abundance and ability to react with 33‐mer antibodies of edited lines

The NGS data provide valuable insights into the abundance of CD epitopes in the edited lines compared to the wild‐type (WT). The NGS amplicon encompasses a region containing the p31‐43 peptide, the 33‐mer region and the DQ2.5_glia_α3 epitope. To assess the impact on immunogenicity due to the excision of the 33‐mer peptide and its variants, the abundance of the CD epitopes in the edited wheat lines was calculated.

**Figure 5:**
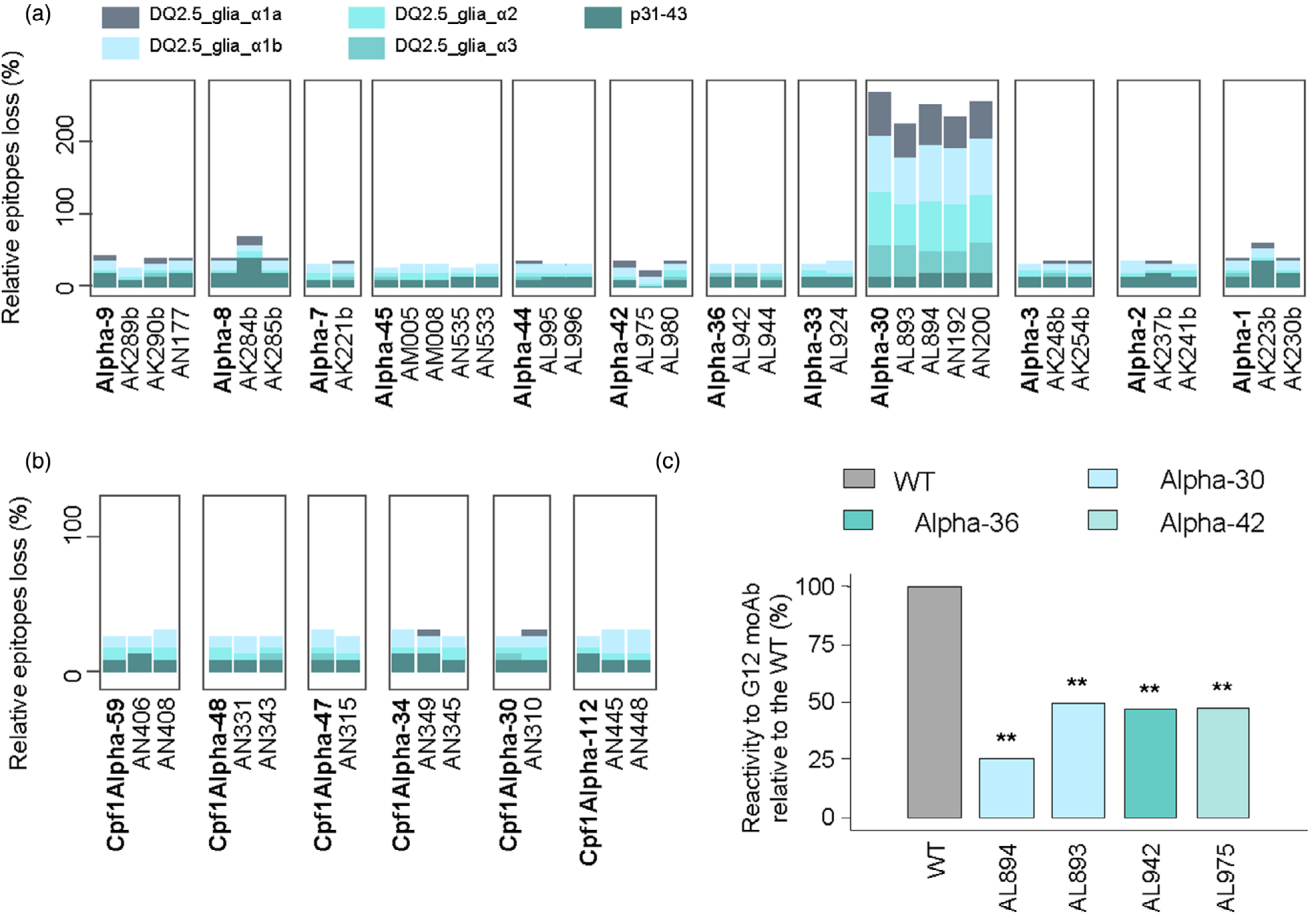
DQ epitopes and reactivity to G12 moAb characterization in edited plants. Frequency (%) of relative loss of CD α‐gliadin epitopes and p31‐43 peptide in (a) Cas9 and (b) Cas12a plants. (c) Percentage of reduction of the reactivity to G12 moAb in CRISPR plants compared to the wild‐type (BW208). ** *P* < 0.01.

As shown in Figure 5a, the Alpha‐30 line and its progeny showed a significant reduction of CD‐immunogenic‐related WT epitopes. Additionally, two T1 lines, AK223b (Alpha‐1) and AK284b (Alpha‐8), both transformed with the pSSLAlpha9 plasmid, presented the highest reduction of the p31‐43 peptide, distinguishing them from their siblings (Figure 5a). Notably, plants transformed with the pSSLCpf1‐3Alpha‐1 construct did not show a high removal of CD epitopes. However, these plants were notably characterized by the absence of DQ2.5_glia_α1a in many instances (Figure 5b). The relative frequency of CD epitope loss was calculated based on the relative abundance of WT amplicons containing them. As amplicons can have multiple hits of the same epitopes in some cases, the cumulative relative abundance can exceed 100% of abundance (as indicated in Figure 5a).

Among the lines screened by amplicon sequencing, four lines were chosen to investigate their ability to react with antibodies raised against the 33‐mer peptide. This selection included the Alpha‐30 progeny (transformed with pSSLAlpha9), the AL942 line (transformed with pSSLAlpha10) and the AL975 line (also transformed with pSSLAlpha9), all displaying excision of the complete 33‐mer peptide. The G12 monoclonal antibody (moAb), which specifically targets the epitopes comprising the 33‐mer peptide (Morón *et al*., 2008), serves as an excellent predictor of immunogenicity due to the elimination of the 33‐mer or its variants. Among the lines tested, line AL894 showed the lowest ability to react with this moAb, while lines AL893, AL942 and AL975 also exhibited significant reductions compared to the WT (Figure 5c). Notably, line AL894, derived from the Alpha‐30 line, achieved a 74.2% excision of the 33‐mer region (Figure 4c) and a strong reduction in the abundance of the CD epitopes (Figure 5a).

### Grain protein content of edited plants

**Figure 6:**
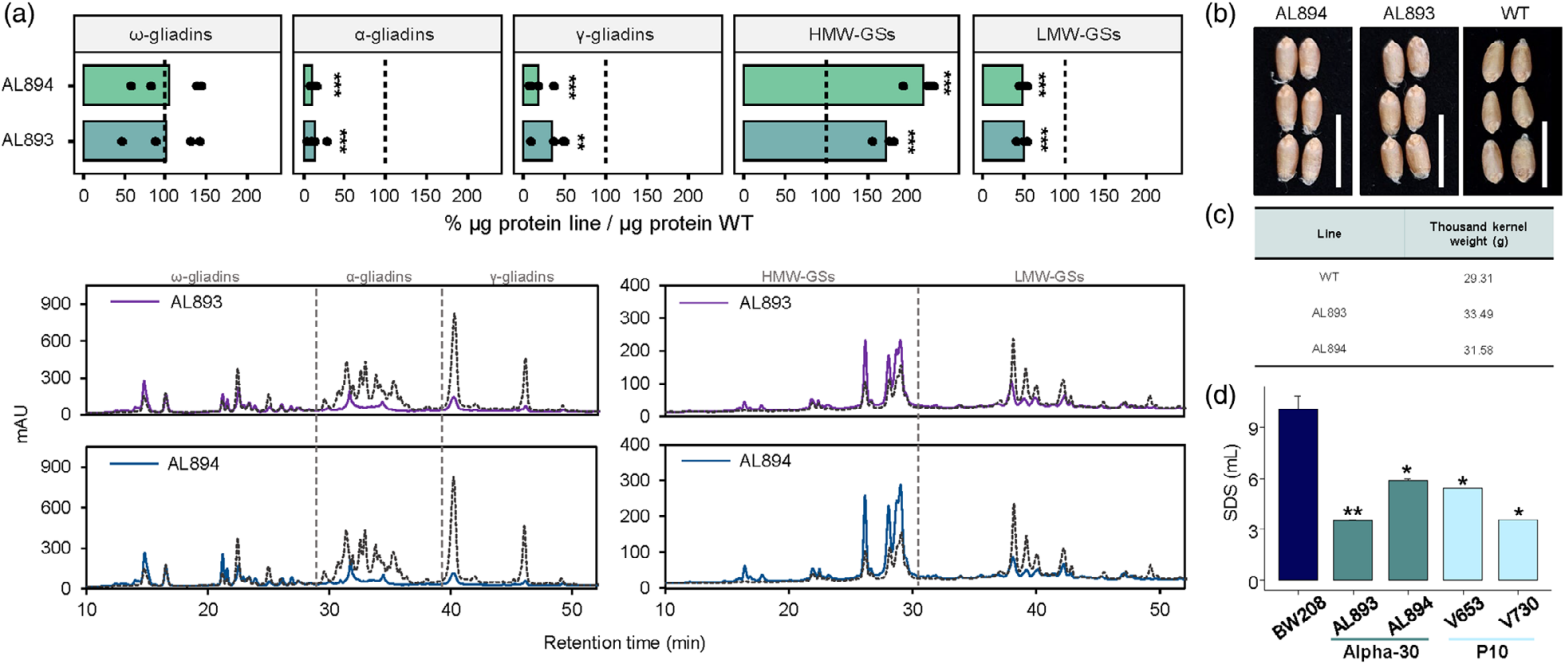
Prolamins, grain shape and SDS sedimentation volume characterization of the Alpha‐30 offspring. (a) Percentage of prolamin fractions content measured by RP‐HPLC in AL893 and AL894 compared to the WT content (equal to 100% in all the cases). The gliadins and glutenins profiles were represented for both lines. The dashed lines represent the WT (BW208) profile. mAU: milli‐absorbance units. (b) Grain shape of AL894 and AL893 lines and the WT (BW208). The white line applies for 1 cm. (c) Thousand kernel weight (g) of the AL893 and AL894 lines and the WT (BW208). (d) SDS sedimentation volume of Alpha‐30 and P10 progeny (obtained in Sánchez‐León *et al*. (2018)). The means and standard errors are represented. *P < 0.05; **P < 0.01; ***P < 0.001.

Both AL893 and AL894 lines, with the highest excision frequencies of the 33‐mer, exhibited a low gliadin content measured by reverse phase‐high performance liquid chromatography (RP‐HPLC). Particularly, both genotypes showed a significant reduction in the α‐ and γ‐gliadin fractions, while the ω‐gliadins remained at the same level as the WT (Figure 6a). These findings align with the A‐PAGE profiles of both lines, which also showed a decrease in both α‐ and γ‐gliadins (Figure 6a). Notably, the HMW‐GSs increased in both lines, mainly in AL894, while the LMW glutenins decreased (Figure 6a). Furthermore, the grain shape of the Alpha‐30 progeny lines remained unchanged compared to the WT, and the thousand kernel weight of AL893 and AL894 showed no difference from the WT. These findings suggest that the genetic modifications did not adversely affect these traits (Figure 6b,c).

These lines were also subjected to the sodium dodecyl sulphate (SDS) sedimentation test. This test is used to indirectly assess the gluten strength of wheat flour by measuring the ability of gluten proteins to form a cohesive network when exposed to SDS. This test is widely accepted in bread wheat breeding programmes as the first predictor of gluten strength and baking quality. The SDS sedimentation test revealed significantly lower volumes (mL) compared to the WT (Figure 6d), similar to the P10‐derived lines obtained previously (Sánchez‐León *et al*., 2018).

The decrease in α‐gliadin content was observed also in plants transformed with pSSLAlpha10 and pSSLCpf1‐3Alpha‐1 (Figure S6). Consistent with the A‐PAGE profiles, these changes were less pronounced in lines transformed with the Cas12a system, where the reduction in α‐ and γ‐gliadins was lower compared to the lines transformed with Cas9 (Figure S6). Interestingly, the lines transformed with pSSLCpf1‐3Alpha‐1 presented slight or strong reductions on some peaks corresponding to ω‐gliadins, mainly in AK687 and AL053 lines (Figure S6).

## Discussion

Wheat prolamins are responsible for several WRDs, which as a whole affect an increasing percentage of the population in Western countries, whose only alternative is to follow a gluten‐free diet for life, with all the health and social consequences that this entails (Marciniak *et al*., 2021). Of all the WRDs, the CD is the best known (Caio *et al*., 2019), and the epitopes present in wheat gliadins, responsible for triggering the immune response, are the target of numerous approaches for the development of therapies and drugs that facilitate the treatment of these pathologies and improve the quality of life of patients (Caio *et al*., 2019). The development of wheat varieties, free of these epitopes, with greatly reduced immunogenicity, could be a very promising alternative as it would avoid the use of drugs and would provide nutritional benefits highly demanded by those affected by these WRDs. However, the development of these varieties is a major challenge due to the complexity of the genome of hexaploid wheat, in which gliadin genes are distributed in multiple loci and chromosomes (Shewry *et al*., 2003).

The use of a single sgRNA (Sánchez‐León *et al*., 2018) resulted in the successful editing of both bread and durum wheat gliadin genes, introducing small/medium‐sized InDels and leading to the knock‐out of a significant proportion of gliadin genes (Sánchez‐León *et al*., 2018, 2024). In the present work, we went a step further by precisely targeting the highly immunogenic regions of α‐gliadins designing a paired sgRNA‐based strategy flanking the regions encoding the complete 33‐mer peptide and its variants to facilitate their excision. Prior research has demonstrated the effectiveness of paired co‐expressed sgRNAs in generating large deletions in single‐copy genes of wheat (Cui *et al*., 2019). Our current research aimed to achieve precise deletions of immunogenic fragments between two sgRNAs and replace them with synthetic DNA exhibiting significantly reduced immunoreactivity potential. The distance between the paired sgRNAs varied from 117 to 75 bp, depending on the construct used for transformation and the targeted α‐gliadin genes. The coding region between the sgRNAs corresponds to a highly variable part in the gene family: genes containing the 33‐mer exhibit longer fragments, whereas those lacking DQ epitopes possess shorter ones.

InDels were analysed using the bioinformatics‐based ampAnalysis software for identifying CRISPR mutations in multi‐copy gene families. In both protoplasts and plants, the two Cas9 constructs effectively induced InDels in the α‐gliadin genes, mainly resulting in small to medium deletions. The most common deletion, −36 bp, was also observed in a previous study using sgAlpha2 (Guzmán‐López *et al*., 2021a), which had also one of the highest editing efficiencies. Notably, its paired sgRNAs in pSSLAlpha9 and pSSLAlpha10 (sgAlpha9 and sgAlpha11, respectively) showed slightly different efficiencies. Additionally, the present work employed the Cas12a system to edit the α‐gliadin gene family, as it expands the potential genomic applications in wheat. LbCas12a was previously reported to be capable of inducing DSBs in the wheat genome; however, its efficiency was lower than that of Cas9 (Wang *et al*., 2021). While genome editing efficiency with LbCas12a in other cereals has been comparable to that observed for Cas9 (Li *et al*., 2018), the sgRNAs efficiency of pSSLCpf1‐3Alpha‐1 was lower than that of the Cas9 system, and protoplasts transformed with this plasmid showed a slightly lower proportion of edited α‐gliadins. Despite these slight differences in efficiency, both Cas9 and Cas12a successfully accomplished the excision and replacement of the highly immunogenic region of the α‐gliadins.

The excision of the 33‐mer and its variants was achieved in protoplast and plant systems with Cas9 and Cas12a, although the latter presented lower efficiencies than the Cas9 editing tool. The variation in the editing efficiencies, along with the fact that not all α‐gliadin genes have all the sgRNA binding sites, suggests that multiple editing events, including large deletions covering paired sgRNAs, would be infrequent. In the same way, the efficiency of the three sgRNAs of the pSSLCpf1‐3Alpha‐1 construct differed, which implies that sgRNA‐to‐sgRNA deletions will be found at low frequency. The most frequent type was the removal of the complete 33‐mer, containing six overlapping epitopes and one epitope downstream (Ozuna *et al*., 2015), followed by the variant containing five DQ epitopes. The most outstanding achievement was in plants, where 74.2% of the complete 33‐mer was removed from a T1 plant edited with Cas9. The 33‐mer excision comprised an in‐frame large deletion of 129 bp, resulting in an α‐gliadin protein lacking the most immunogenic region. This plant and its sibling—with 69.3% of 33‐mer excision—came from the Alpha‐30 line and presented a reduced reactivity to G12 moAb, reached in the 33‐mer region (Morón *et al*., 2008). The Alpha‐30 progeny exhibited a reduced number of DQ epitope hits in the α‐gliadins, reaching values of 200% of relative loss of α‐gliadin DQ2.5 epitopes plus the p31‐43 peptide, and a strong reduction of α‐ and γ‐gliadin proteins. These lines present one of the most interesting options for T‐cell proliferation tests, as their unique α‐gliadins profile with almost the total loss of the 33‐mer converts them into a great starting point for obtaining low‐immunogenic wheat suitable for CD patients. These lines also showed an increase in the HMW glutenins, likely due to a compensation mechanism previously reported in CRISPR/Cas and RNAi wheat lines (Marín‐Sanz and Barro, 2022; Pistón *et al*., 2013; Sánchez‐León *et al*., 2018). Additionally, a significant decrease in LMW‐GSs was noted in both lines, which may be attributed to the potential co‐regulation of gliadins and LMW glutenins by a network of transcription factors (Marín‐Sanz and Barro, 2022). This complex and interconnected prolamins regulation network may also explain the ω‐gliadin reduction in some plants edited with the pSSLCpf1‐3Alpha‐1 construct. Given the crucial role of glutenins in the bread‐making quality (Payne, 1987), AL893 and AL894 lines were subjected to the SDS sedimentation test, as this property correlates with SDS volumes (Carter *et al*., 1999). Both AL893 and AL894 exhibited significant reduction in SDS values compared to the WT, aligning with findings from previous wheat lines with substantial gliadins reduction achieved through CRISPR and RNAi technologies (Gil‐Humanes *et al*., 2010; Sánchez‐León *et al*., 2018). Notably, some RNAi lines demonstrated tolerance to over‐mixing and enabled the production of bread with baking and sensory properties comparable to those of standard wheat flour (Gil‐Humanes *et al*., 2014a,b). Although further assays are required to confirm these properties in the Alpha‐30 progeny, these findings suggest that both AL893 and AL894 may exhibit bread‐making attributes similar to those observed in these RNAi lines.

CRISPR tools offer other opportunities to reach precise genome editing, taking advantage of the NHEJ pathway to get targeted insertions in specific DNA binding sites. The NHEJ pathways had higher efficiencies compared to the HDR‐mediated modifications (Chen *et al*., 2019), converting the first one to more suitable to obtain frequent targeted insertions. This was reported in rice, where outcomes such as introducing transcription activator‐like effector binding sites into the promoter of bacterial blight resistance gene and a translational enhancer in a salt tolerance locus were obtained using chemically stabilized dsODNs (Kumar *et al*., 2023; Lu *et al*., 2020). In this work, we leveraged of paired sgRNA designs flanking the immunogenic regions of α‐gliadin genes to excise the entire fragment and replace it with a synthetic dsODN. We co‐transformed protoplast and plant systems with a chemically stabilized dsODN based on the redesign of the region containing the 33‐mer. In this case, nucleotide changes coding for new enzyme cleavage sites and reducing epitope toxicity were introduced in the 33‐mer template to create a dsODN capable of reducing the immunoreactivity of α‐gliadins. Certain substitutions of the 9‐mers comprising the DQ2.5 epitopes abolished immunogenicity: the substitution of Pro (P) to Ser (S) at position eight of the 9‐mer and the replacement of Gln (Q) to His (H) in the DQ2.5 epitopes domain lead to the reduction of the immunoreactivity of α‐gliadins (Ruiz‐Carnicer *et al*., 2019). Additionally, the substitution of Gln (Q) to Lys (K) at position six of the DQ2.5_glia_α1a reduced drastically the Interferon γ (IFN‐γ) ELISPOT response compared to the original peptide (Anderson *et al*., 2006). Furthermore, the introduction of amino acid changes which promotes the appearance of new digestion enzymes' cleavage site (Figure S3) will favour the digestibility of α‐gliadin peptides in the human small intestine, where the main protein degradation process takes place (Gray, 2011).

Substitutions of the high immunogenic regions were achieved in both Cas9 and Cas12a systems reaching values of more than 12% of targeted insertions with Cas9 plasmids. Based on previous research, all dsODNs were phosphorylated at the 5′ to facilitate NHEJ, bearing phosphorothioate linkages at both 5′ and 3′ ends to prevent degradation, as those modifications presented positive effects on targeted insertions in mammalian and plant cells previously (Lu *et al*., 2020; Tsai *et al*., 2015). Based on the study of Kumar *et al*. (2023), we designed both dsODN with BE and sticky ends to compare the performance of not directionally and directionally controlled dsODN insertions, respectively. In the case of BE dsODN, we observed that the targeted insertions were in forward and reverse directions interchangeably in protoplasts transformed with the Cas9 system, mainly in the forward direction. There is now evidence that Cas9 can introduce a 1‐nucleotide 5′ overhang (Hussmann *et al*., 2021), leading to a study of the improvement of direction control in targeted insertions in Cas9 systems (Kumar *et al*., 2023). These insights lead us to use OV dsODN, where the targeted insertion occurs in the forward direction predominantly. However, some protoplasts transformed with the Cas9 system and the OV dsODN presented low‐frequency targeted insertions in the reverse direction. This may be because in this type of genes, the InDels do not always conform to the expected product 5′ of the PAM region, leading to a wider range of insertion sites and the loss of controlled direction of targeted insertions. As Kumar *et al*. (2023) ventured, the overhang‐producing Cas12a system could directionally control targeted insertions, as Cas12a can introduce 5 or 4‐nucleotide 5′ overhang (Zetsche *et al*., 2015). In this study, we obtained the evidence that forward dsODN 5 nt overhang insertions with direction control occur in wheat protoplasts, combined with a previous sgRNA‐to‐sgRNA excision, to introduce precise replacements in the genome. However, the dsODN OV used in the Cas12a system exhibited lower efficiencies than the Cas9‐transformed protoplasts. Overall, these results showed the successful replacement of the immunogenic fragments contained between paired sgRNAs with non‐immunogenic sequences in both Cas9 and Cas12a systems. However, the efficiency of the targeted insertion remains low compared to not‐targeted insertions or deletions. Moreover, the differences in the sgRNA efficiencies of the same plasmid decrease the probability of achieving the complete excision and followed substitution. Probably due to the low efficiency of dsODN insertions in protoplasts, we did not find targeted insertions in plants.

Achieving substitution in regions of wheat with high DQ epitope density marks a significant advancement in precision genome editing. Although the dsODN insertion was not achieved in wheat plants, the successful removal of the 33‐mer from α‐gliadin genes transformed these sequences into in‐frame gliadins with no potential for adaptive immunoreactivity. This represents an important step forward in the redesigning of wheat gliadins, aimed to remove immunogenicity but keeping functionality. However, as the excision of the 33‐mer coding region resulted in novel shorter proteins, further studies of stimulation assays using PBMCs from celiac patients are necessary to fully characterize their immunoreactivity and functionality. In addition, to improve the targeted replacement of immunogenic α‐gliadin sequences in wheat, future approaches could explore several alternative strategies. Prime Editing (PE), although still inefficient in wheat (Zong *et al*., 2022), may enable precise sequence substitutions. Recent developments in paired PE, where two pegRNAs are used to introduce complementary edits at both ends of a target sequence (Anzalone *et al*., 2022), could provide an alternative to replacing the 33‐mer with a non‐immunogenic sequence while maintaining the correct reading frame. Transposase‐assisted genomic editing represents another promising approach, as it takes advantage of mobile genetic elements to facilitate precise sequence substitution (Liu *et al*., 2024).

## Experimental procedures

### Plasmid design and construction

For the CRISPR constructs design, the 45 BW208 α‐gliadin gene sequences characterized in Sánchez‐León *et al*. (2021) were used. Conserved regions on these sequences by alignments were used for sgRNA protospacer designs. The on‐target potential activities of the protospacers predicted by Geneious v2020.1.1 (Biomatters Ltd., Auckland, New Zealand), and the off‐target hits throughout the hexaploid wheat genome (Appels *et al*., 2018) were considered to select the best ones, maximizing the number of α‐gliadin genes targeted in BW208.

The Golden Gate assembly protocol was used to construct the Cas9 and Cas12a vectors (Čermák *et al*., 2017). Each protospacer was cloned into pMOD‐B2518 (Addgene #91075) or pMOD‐C2518 (Addgene #91087) modules via synthetic oligo annealing with complementary overhangs to the Esp3I restriction enzyme present in both entry modules 5′ of the sgRNA scaffold (Figure 1c). For the Cas9 constructs, paired sgRNAs were designed to flank the highly immunogenic region of α‐gliadin genes (Figure 1d). In the Cas12a construct, two of the three sgRNAs were designed to flank this region, while the third targeted the first DQ epitopes within this area (Figure 1d). Two nucleases, codon optimized for monocots, were used in this study: SpCas9 and LbCas12a, both driven with the ZmUbi promoter (Figure 1c). Both were included in an intermediate pMOD‐A1110 module subcloned into a final backbone. This module contains also nuclear localization signals (NLSs) and an octopine synthase terminator (OCSt) (Figure 1c). To check the construction of the CRISPR vectors, the whole plasmids were sequenced by Oxford Nanopore long‐read sequencing (Plasmidsaurus, Oregon, US).

### dsODN design and construction

The dsODNs were designed based on the 33‐mer template introducing SNPs to change the amino acid composition of these fragments, adding new digestion enzyme cleavage sites, and substituting amino acids from the 9‐mer core of the DQ epitopes to reduce their immunoreactivity (Figures S2 and S3). Depending on the cut site of the Cas9/Cas12a and sgRNAs used, different sequences for the dsODNs were designed to keep the sequences in‐frame (Figure S2). Sequences were synthesized as single‐strand oligos by Integrated DNA Technologies (IDT, 1710 Commercial Park, Coralville). Additionally, all single‐stranded oligos were phosphorylated at the 5′ to facilitate NHEJ and bore phosphorothioate linkages at both 5′ and 3′ ends (Figure S2a,b) to prevent degradation in plant cells (Kumar *et al*., 2023; Lu *et al*., 2020; Tsai *et al*., 2015). dsODNs were obtained by annealing equimolar concentration of single‐stranded oligos at 94 °C for 2 min, cooling at RT for 1 h and adding 80 μL of duplex buffer (IDT). dsODNs were then checked on 1% agarose gels.

### Protoplasts isolation and transformation

*Triticum aestivum* (cv. BW208) leaves from ~15‐day‐old *in vitro* seedlings were used for protoplasts' isolation and transfection as described by Weiss *et al*. (2020), with slight modifications. For protoplasts' transfection, between 10 and 300 μmol of dsODN and 10 μg of Cas plasmids were incubated with 150 000 protoplasts in a 20% (w/v) polyethylene glycol (PEG) solution (pH 5.7). Transfected protoplasts were washed twice and incubated in W5 buffer (150 mM NaCl, 125 mM CaCl_2_, 5 mM KCl_2_ and 2 mM MES pH 5.7) at 25 °C for 48 h in the dark. Transformation efficiency was monitored through the fluorescence from the GFP reporter gene using the EVOS M5000 fluorescence microscope (Thermo Fisher Scientific Inc., Waltham, MA).

### Plant material, growth conditions and plant transformation

*Triticum aestivum* (cv. BW208) WT genotype was used as the source of immature scutella, which constituted the explants for genetic transformation (Piston *et al*., 2009). The wheat plants were grown in a greenhouse under controlled conditions: a 12 h/12 h day/night photoperiod, a 24 °C/16 °C temperature regime, and 60% relative humidity. The scutellum of each plant was isolated and cultured *in vitro* as described by León *et al*. (2006). The explants were bombarded with 0.6 μm gold particles at 1.5 pmol DNA plasmid/mg gold particles. The dsODN was delivered at 75 and 150 pmol/mg gold. The scutella were regenerated *in vitro* in 2 mg/L of PPT medium, where transformed plants were selected as previously described (Piston *et al*., 2008), using the *bar* gene under the *Panicum virgatum* ubiquitin promoter (PvUbi2). The transformed plants were transferred to pots in the greenhouse under the same conditions mentioned above, allowing them to reach maturity.

### DNA extraction, PCR and NGS sequencing

The DNA was extracted from leaves of plants and protoplasts following the CTAB protocol (Murray and Thompson, 1980), and its concentration was measured by NanoDrop ND‐1000 (Thermo Fisher Scientific, Waltham, MA). The α‐gliadin genes were amplified with the primers aGli900F1 (5′‐GTTAGAGTTCCAGTGCCACAA‐3′) as forward and 33mer1R2‐Ok (5′‐GGTTGTTGTGGTTGCGRATA‐3′) as reverse, as described previously (Ozuna *et al*., 2015). The amplicons cover the p31‐43 and 33‐mer peptides in the coding part of the first repetitive domain (Marín‐Sanz *et al*., 2023). The amplicon sequencing of the protoplast samples was carried out by GENEWIZ (2X300 bp, Azenta Life Sciences) with the MiSeq system (https://www.illumina.com/), while the amplicons from the plant samples were sequenced by Fundación Parque Científico de Madrid (Cantoblanco, Madrid, Spain) by the NextSeq system (2 × 280 bp, https://www.illumina.com/). The length of the amplicons was checked using the Agilent 2100 Bioanalyzer system (Agilent Technologies, Santa Clara, CA).

### Cas9 and Cas12a genes detection by PCR

The presence of the *Cas9* gene was detected by PCR as described in Sánchez‐León *et al*. (2018). For the detection of the *Cas12a* gene, a 25 μL volume reaction was used, containing 300 ng of total DNA, 320 μm dNTP mix, 400 nm of forward and reverse primers and 0.65 units of Taq DNA polymerase (Biotools, Madrid, Spain). The forward primer LbCpf1‐F1 (5′‐AAGAACGACGCTGTTGTTGC‐3′) and the reverse primer LbCpf1‐R1 (5′‐GCATGAAGAGTTCAGCCCCT‐3′) were used for the PCR. The PCR conditions were as follows: an initial denaturation at 94 °C for 4 min, followed by 35 cycles of 94 °C for 30 s, 58 °C for 40 s and 72 °C for 1 min, with a final extension at 72 °C for 7 min.

### NGS amplicon analysis

For the analysis of NGS amplicon deep sequences, a comprehensive pipeline was developed to cover the entire process from amplicon assembly to InDels detection. This pipeline was implemented through a custom software named ampAnalysis, which encompasses four main steps: (i) the obtention of samples' Amps, (ii) the trimming of non‐α‐gliadin Amps, (iii) the alignment of Amps to the BW208 Amps database and (iv) the InDels/ODN analysis (Figure S1). The ampAnalysis code is available at https://github.com/MiriamMarinS/ampAnalysis.

For the obtention of sample Amps, raw reads were processed using Usearch v9.2.64 (Edgar, 2010), with steps including merging (−fastq_mergepairs), low‐quality filtering (−fastq_filter), dereplication (−fastqx_uniques) and denoising (−unoise2), using option values from Bayesian optimization performed in previous research (Guzmán‐López *et al*., 2021a). The resulting denoised amplicons, termed Amps, had their abundance quantified by searching and mapping raw reads to the Amps database (‐search_exact). To filter for α‐gliadin Amps, 3161 α‐gliadin sequences from the NCBI nt database (visited on June 2023) were downloaded (https://www.ncbi.nlm.nih.gov/). The ultra‐fast software MMSeqs2 (Steinegger and Söding, 2017) was used to match the Amps against this database (Steinegger and Söding, 2017), removing any non‐matching sequences to eliminate off‐target products.

Before InDels and dsODN analysis, each Amp sequence was aligned to the BW208 Amps database to identify the closest reference sequence. Two alignment strategies were used: (i) local alignment with BWA‐MEM (Li, 2013), a Burrow‐Wheeler Aligner for short reads, and (ii) global alignment with BBmap v39.01 (Bushnell, 2014).

All these steps were executed using the ampAnalysis software on an HPC Cluster with 24 Bull x440 nodes, with 192 GB of RAM per node, totaling 960 cores (Advanced Computing Unit (UCAS), University of Córdoba, Spain).

#### InDels and dsODN insertion analysis

The sgRNAs were searched within the BW208 Amps to identify their positions if the seed sequence (from PAM to 12 bp 5′ of the sequence) and the PAM motif had a perfect match. The positions of InDels for each sample Amp, as obtained from the alignment results, were then compared to the relative reference positions of the sgRNAs to classify these mutations as putative CRISPR editions. Given the repetitive nature of the α‐gliadin genes, the initial positions of the InDels did not always overlap perfectly with the sgRNAs positions. To address this, realignment functions were implemented to refine these sections and reduce the number of false‐negative edits. The frequencies of InDels were then calculated based on the Amps abundance data obtained in the initial step of the pipeline. The insertion of dsODN fragments was analysed using alignment results, considering the positions of the sgRNAs in the reference Amp sequences.

### Prolamin characterization and SDS sedimentation test of edited plants

Different prolamin fractions were analysed following the protocol described in Gil‐Humanes *et al*. (2012). The gliadin proteins were extracted and separated by A‐PAGE from half‐seed samples previously milled, preserving the embryo for line propagation.

For gliadin and glutenin quantification, 100 mg of flour was used for the sequential protein extractions, and measurements were conducted by RP‐HPLC following the protocol described in Pistón *et al*. (2011).

The SDS sedimentation test volume was determined as described in Williams *et al*. (1988), with two technical replicates analysed for each sample.

### ELISA G12 monoclonal antibody (moAb) analysis

The reactivity to G12 moAb was measured using the GlutenTox ELISA Rapid G12 kit (Hygiena, CA). Gliadin extraction and the ELISA assay were repeated over 3 days, with each assay performed in duplicate. These analyses were conducted by the Laboratorio de Análisis de Gluten UPV/EHU – GLUTEN3S from the University of the Basque Country (Vitoria‐Gasteiz, Spain).

### Statistical analysis

The comparison of samples was conducted following the normalization assumption test using Kruskal–Wallis for non‐parametric data and one‐way ANOVA for parametric data. Pairwise mean comparisons were carried out using two‐sided *t*‐tests or Welch's *t*‐tests for parametric data, and two‐sided Mann–Whitney–Wilcoxon tests for non‐parametric data. All the statistical analyses were performed using R software (R Core Development Team, 2020).

## Accession numbers

The datasets presented in this study can be found in online repositories of the National Center for Biotechnology Information (NCBI) database under the identifier PRJNA1170852 (BioProject).

## Author contributions

F.B. conceived and designed the project. F.B., D.F.V. and C.G.S. designed the protoplast experiments, and F.B. performed the assays. M.M.‐S. conducted the data analyses, coded the sequences analysis software and interpreted the data. S.S.‐L. designed the plasmids. M.H.G.‐L. conducted the protein experiments. M.M.‐S. and F.B. wrote the draft manuscript. M.M.‐S., F.B., C.G.S. and D.F.V. reviewed the manuscript. F.B. acquired the funding. All authors approved the final manuscript.

## Conflict of interest

The authors declare that the research was conducted in the absence of commercial or financial relationships that could be construed as a potential conflict of interest.

## Supporting information


**Figure S1** InDels analysis workflow used in ampAnalysis software. InDels, 33‐mer excision and dsODN insertion analysis pipeline: starting from amplicon processing to sequence alignments using external tools, and the implementation of in‐house algorithms to detect CRISPR mutations.

**Figure S2** Characterization of dsODNs sequences. (a) Characteristics of dsODNs used in the present work, the forward and reverse sequences were included. The asterisks indicate the position of the phosphorothioate linkage at 5′ and 3′ ends. (b) Scheme of double‐stranded blunt‐ends and 1 or 5 bp overhangs dsODNs. All of them are 5′‐phosphorylated. (c) The abundance of α‐gliadin Amps with at least two different sgRNAs per plasmid and Amp type: Amps with potential dsODN substitution. The means for the wild‐type (BW208) and the standard errors are represented.

**Figure S3** Probability of potential cleavage sites of dsODN fragments. The probability of potential cleavage sites of enzymes in (a) the fragment extracted from sgAlpha2 to sgAlpha9 containing the complete 33‐mer (based on the Alpha7 type Amp protein), (b) the dsODN‐Cas9 51 bp, (c) the dsODN‐Cas9 75 bp, (d) the dsODN‐Cpf1 51 bp, and (e) the dsODN‐Cpf1 75 bp protein sequences. The probability of potential cleavage is calculated for chymotrypsin and trypsin enzymes (0%–100%) with the PeptideCutter software from Expasy (https://web.expasy.org/peptide_cutter/). The model for the probability of cleavage prediction is not available for the other enzymes, thus a dichotomous variable (0% or 100%) is used to indicate the cleavage positions for those enzymes. The 33‐mer peptide is highlighted in purple in (a), and the amino acid changes in dsODN proteins compared to the 33‐mer (b–e) are marked in red.

**Figure S4** Gliadin profiles of Cas9 plants by A‐PAGE. A‐PAGE gliadin profiles of plants transformed with Cas9‐based pSSLAlpha9 and pSSLAlpha10 constructs. BW208 represents the WT gliadin profile.

**Figure S5** Gliadins profiles of Cas12a plants by A‐PAGE. A‐PAGE gliadin profiles of plants transformed with Cas12a‐based pSSLCpf1‐3Alpha‐1 construct. The red arrows and boxes indicate the absence of bands, the position of softer bands, or the appearance of new bands. BW208 represents the WT gliadin profile.

**Figure S6** Gliadin profiles of edited plants by RP‐HPLC. Gliadins profile by RP‐HPLC of plants transformed with pSSLAlpha9, pSSLAlpha10, and pSSLCpf1‐3Alpha‐1 constructs. The dashed lines represent the WT (BW208) profile. The parental line (T0) is indicated for each line. mAU, milli‐absorbance units.

**Table S1** List of independent protoplasts isolations transformed with pSSLAlpha9, pSSLAlpha10, and pSSLCpf1‐3Alpha‐1. BE, blunt end; OV, overhang.

## Data Availability

The data that support the findings of this study are openly available in BioProject at https://www.ncbi.nlm.nih.gov/bioproject/PRJNA1170852, reference number PRJNA1170852.
