# Supplementary figures and images for: Cas9‐ and Cas12a‐mediated excision and replacement of the celiac disease‐related α‐gliadin immunogenic complex in hexaploid wheat
Source: Plant Biotechnol J. 2025 Jun 15;23(9):3798–813. doi: 10.1111/pbi.70200 (PMC12392967; doi:10.1111/pbi.70200)

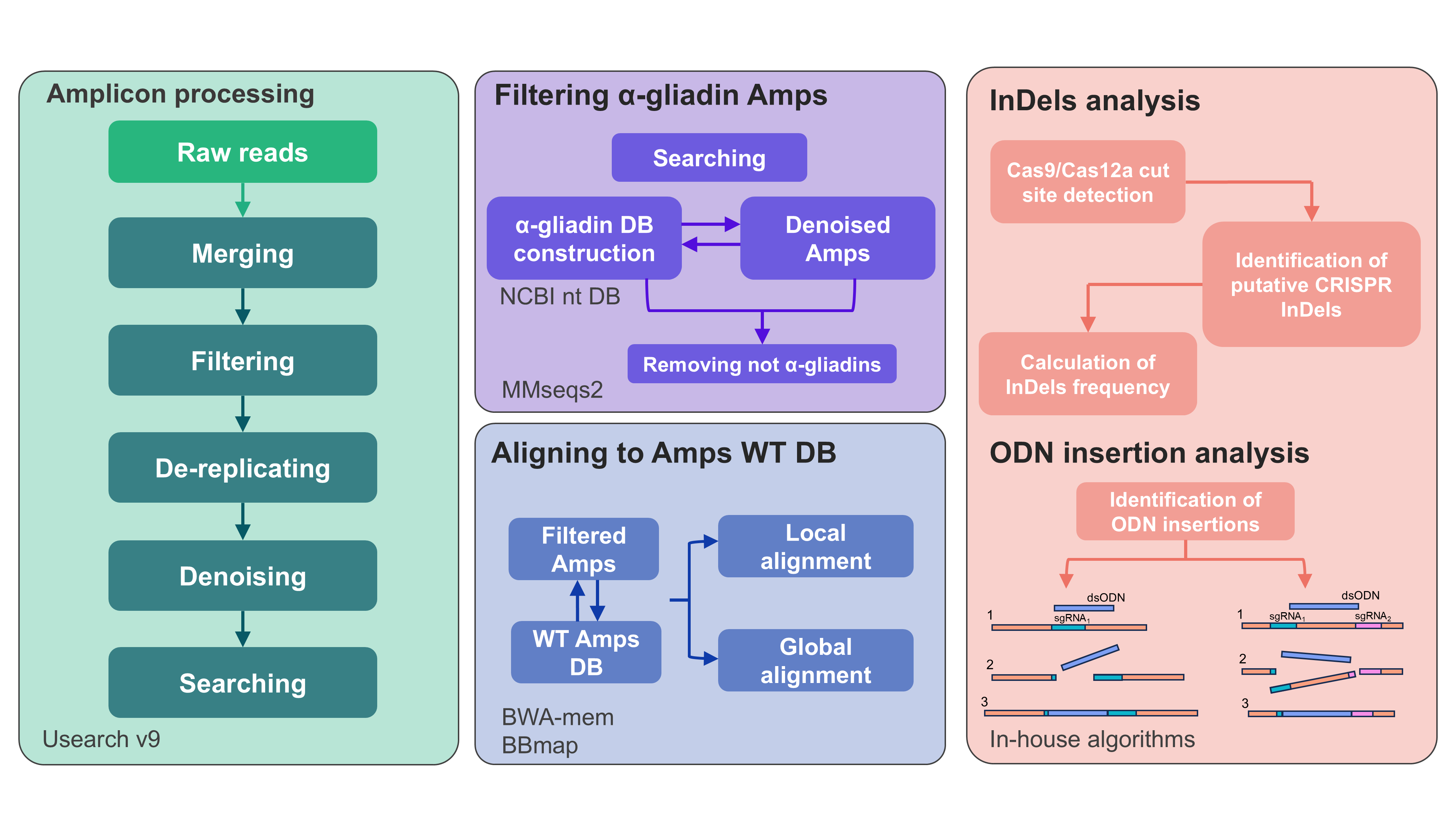

Supplement: Supplementary file 1 — Figure S1 InDels analysis workflow used in ampAnalysis software. InDels, 33‐mer excision and dsODN insertion analysis pipeline: starting from amplicon processing to sequence alignments using external tools, and the implementation of in‐house algorithms to detect CRISPR mutations. [file PBI-23-3798-s004.tif]

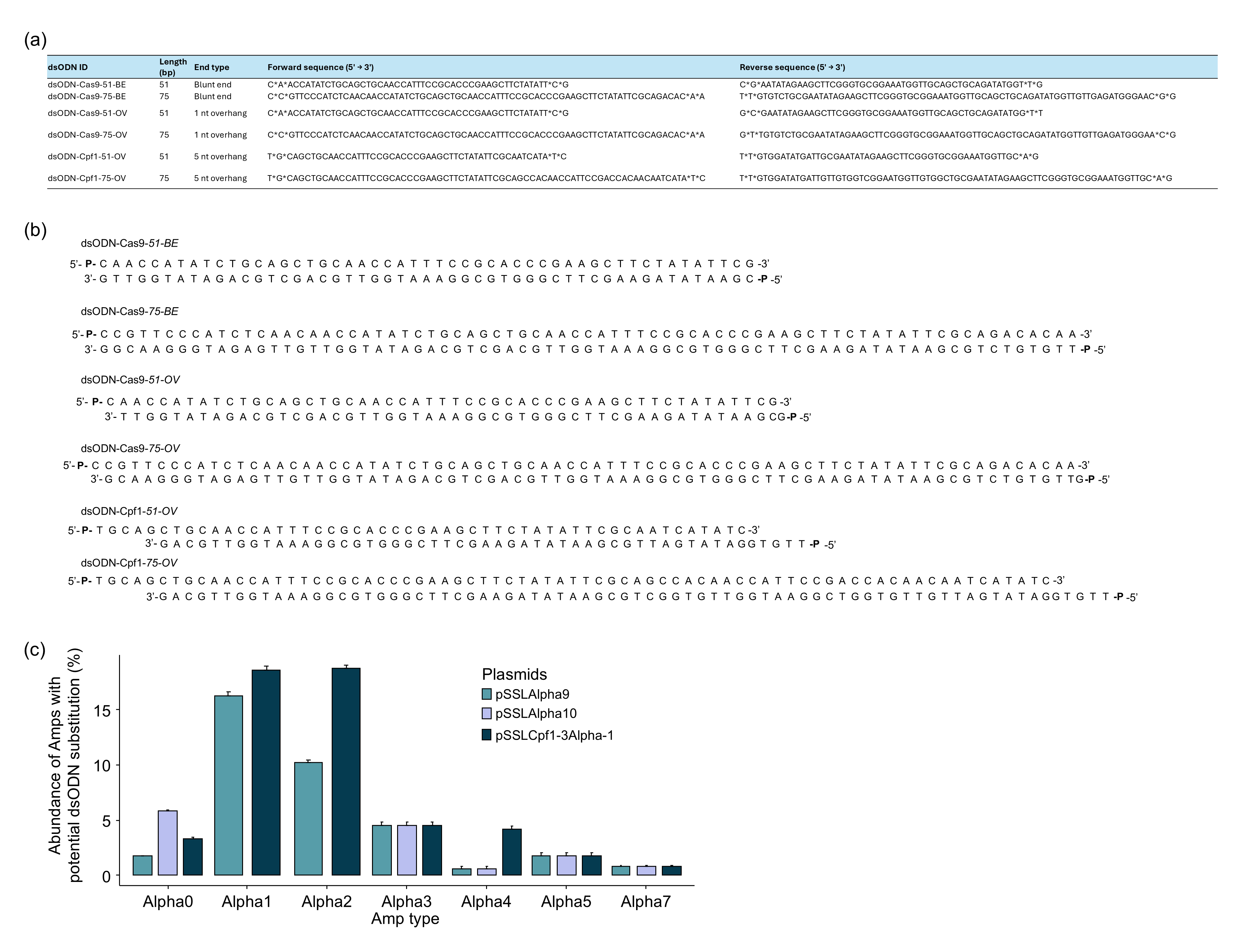

Supplement: Supplementary file 2 — Figure S2 Characterization of dsODNs sequences. (a) Characteristics of dsODNs used in the present work, the forward and reverse sequences were included. The asterisks indicate the position of the phosphorothioate linkage at 5′ and 3′ ends. (b) Scheme of double‐stranded blunt‐ends and 1 or 5 bp overhangs dsODNs. All of them are 5′‐phosphorylated. (c) The abundance of α‐gliadin Amps with at least two different sgRNAs per plasmid and Amp type: Amps with potential dsODN substitution. The means for the wild‐type (BW208) and the standard errors are represented. [file PBI-23-3798-s005.tif]

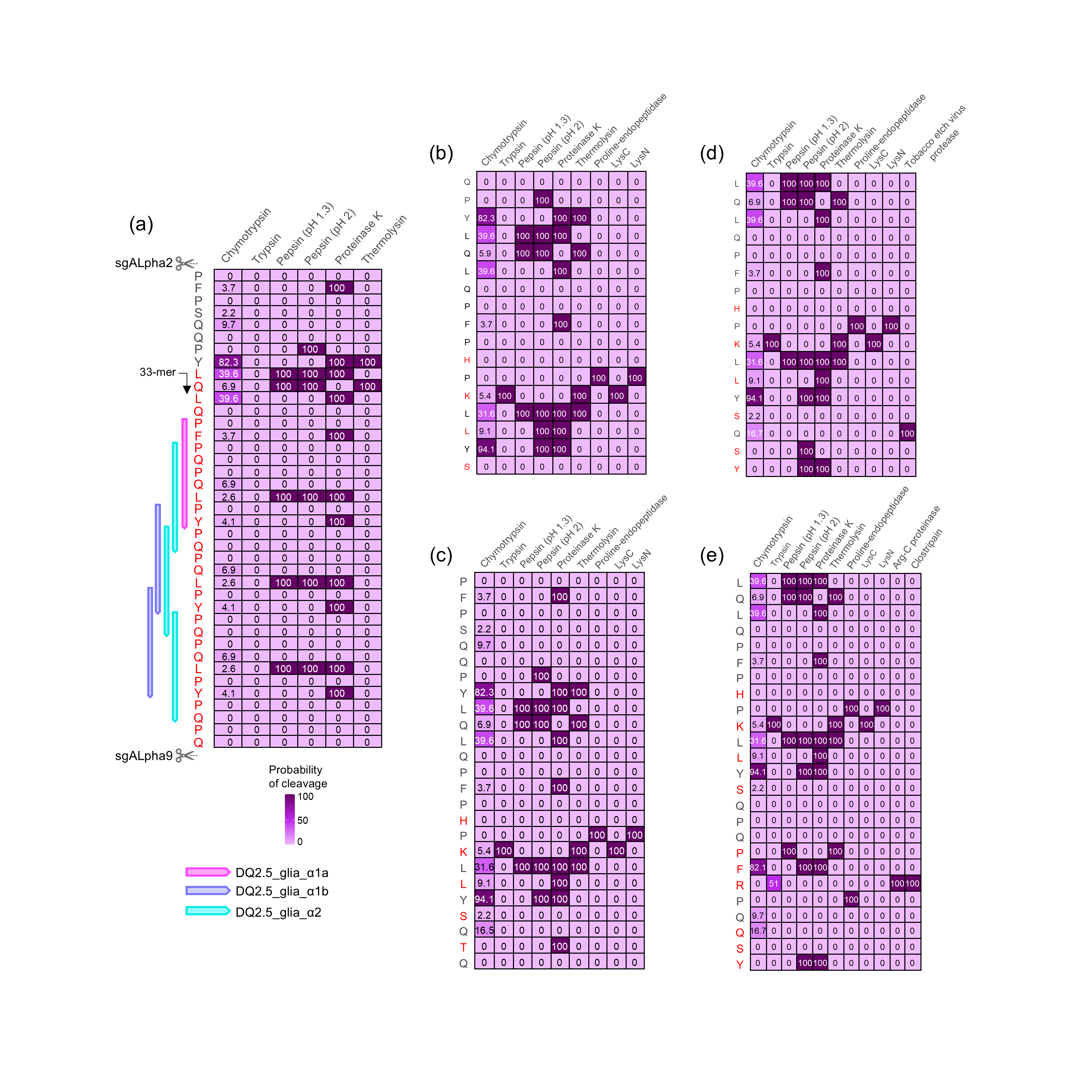

Supplement: Supplementary file 3 — Figure S3 Probability of potential cleavage sites of dsODN fragments. The probability of potential cleavage sites of enzymes in (a) the fragment extracted from sgAlpha2 to sgAlpha9 containing the complete 33‐mer (based on the Alpha7 type Amp protein), (b) the dsODN‐Cas9 51 bp, (c) the dsODN‐Cas9 75 bp, (d) the dsODN‐Cpf1 51 bp, and (e) the dsODN‐Cpf1 75 bp protein sequences. The probability of potential cleavage is calculated for chymotrypsin and trypsin enzymes (0%–100%) with the PeptideCutter software from Expasy (https://web.expasy.org/peptide_cutter/). The model for the probability of cleavage prediction is not available for the other enzymes, thus a dichotomous variable (0% or 100%) is used to indicate the cleavage positions for those enzymes. The 33‐mer peptide is highlighted in purple in (a), and the amino acid changes in dsODN proteins compared to the 33‐mer (b–e) are marked in red. [file PBI-23-3798-s007.tif]

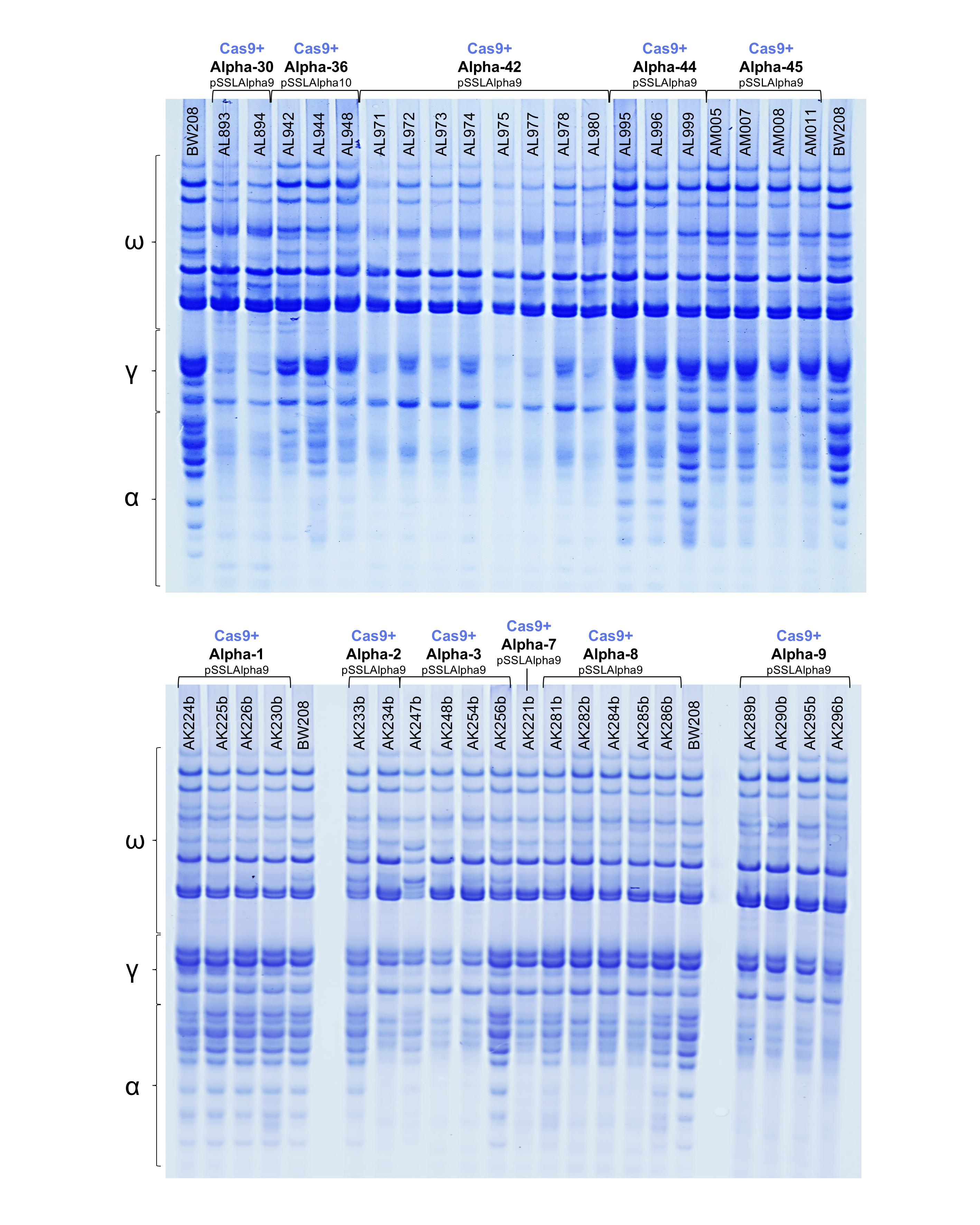

Supplement: Supplementary file 4 — Figure S4 Gliadin profiles of Cas9 plants by A‐PAGE. A‐PAGE gliadin profiles of plants transformed with Cas9‐based pSSLAlpha9 and pSSLAlpha10 constructs. BW208 represents the WT gliadin profile. [file PBI-23-3798-s002.tif]

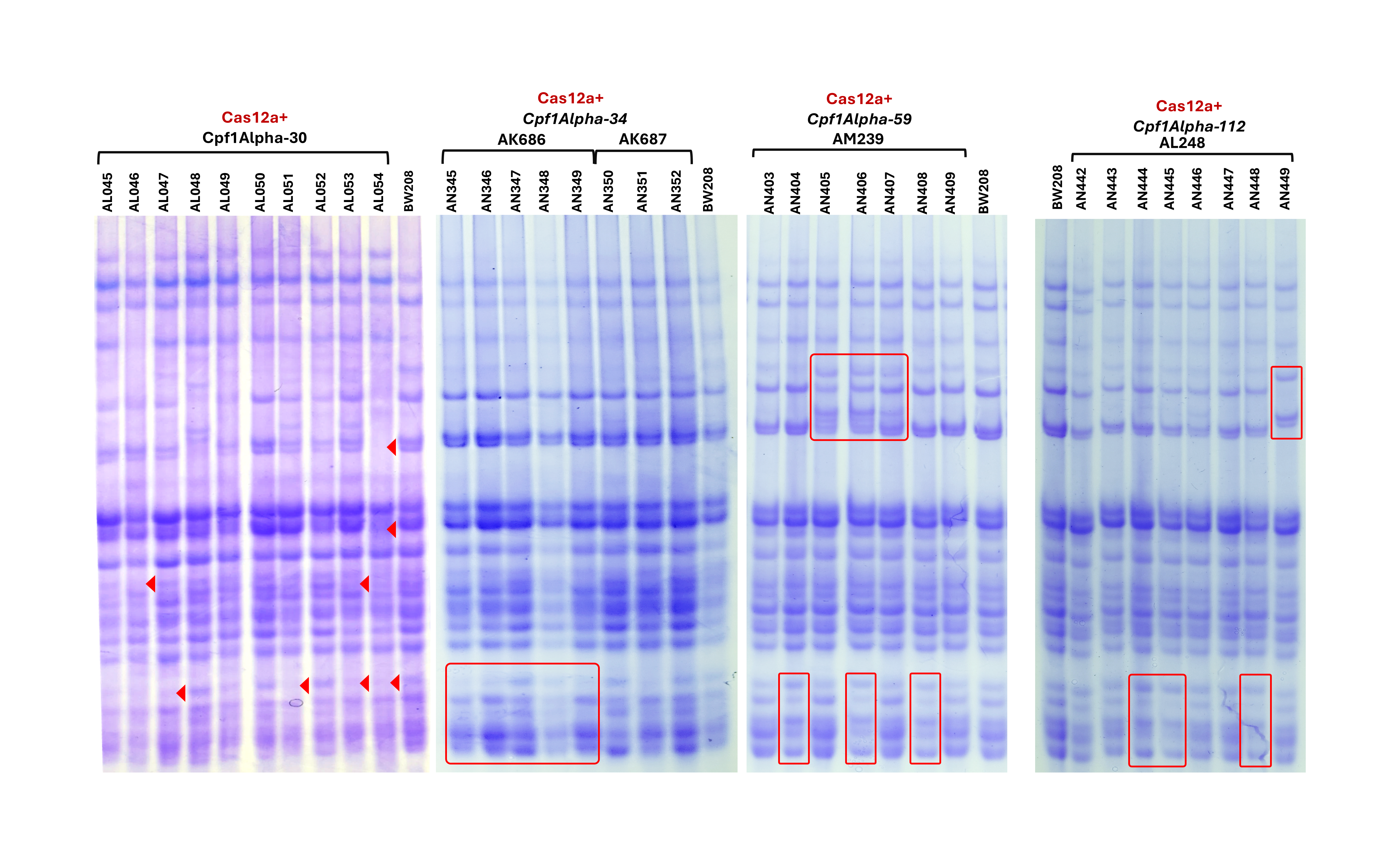

Supplement: Supplementary file 5 — Figure S5 Gliadins profiles of Cas12a plants by A‐PAGE. A‐PAGE gliadin profiles of plants transformed with Cas12a‐based pSSLCpf1‐3Alpha‐1 construct. The red arrows and boxes indicate the absence of bands, the position of softer bands, or the appearance of new bands. BW208 represents the WT gliadin profile. [file PBI-23-3798-s003.tif]

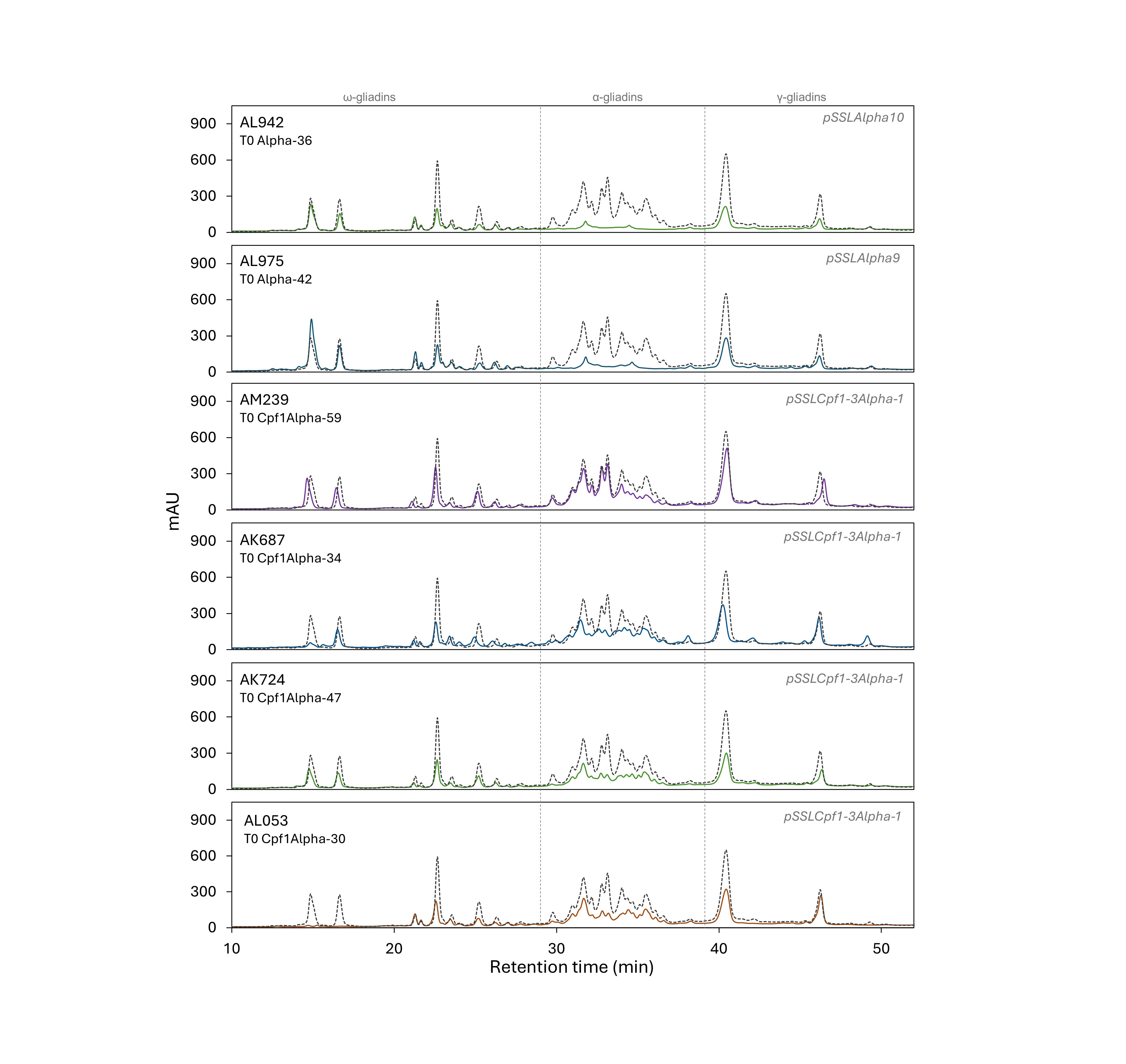

Supplement: Supplementary file 6 — Figure S6 Gliadin profiles of edited plants by RP‐HPLC. Gliadins profile by RP‐HPLC of plants transformed with pSSLAlpha9, pSSLAlpha10, and pSSLCpf1‐3Alpha‐1 constructs. The dashed lines represent the WT (BW208) profile. The parental line (T0) is indicated for each line. mAU, milli‐absorbance units. [file PBI-23-3798-s006.tif]
