# Supplementary material for: Cas9‐ and Cas12a‐mediated excision and replacement of the celiac disease‐related α‐gliadin immunogenic complex in hexaploid wheat
Source: Plant Biotechnol J. 2025 Jun 15;23(9):3798–813. doi: 10.1111/pbi.70200 (PMC12392967; doi:10.1111/pbi.70200)
Supplement: Supplementary file 7 — Table S1 List of independent protoplasts isolations transformed with pSSLAlpha9, pSSLAlpha10, and pSSLCpf1‐3Alpha‐1. BE, blunt end; OV, overhang. [file PBI-23-3798-s001.pdf]

Table S1. List of independent protoplasts isolations transformed with pSSLAlpha9, pSSLAlpha10, and pSSLCpf1-3Alpha-1. OV: overhang, BE: blunt end.

Experiment	Sample ID	Plasmid for transformation	dsODN ID	ODN length (bp)	ODN end	ODN concentration (μM)
Exp1	P01	pSSLAlpha9	NA	NA	NA	NA
Exp1	P02	pSSLAlpha9	dsODN-Cas9-51-OV	51	OV	20
Exp1	P03	pSSLAlpha9	dsODN-Cas9-51-OV	51	OV	30
Exp1	P04	pSSLAlpha9	dsODN-Cas9-51-OV	51	OV	10
Exp1	P05	pSSLAlpha9	dsODN-Cas9-51-OV	51	OV	20
Exp1	P06	pSSLAlpha9	dsODN-Cas9-51-OV	51	OV	30
Exp1	P07	pSSLAlpha9	dsODN-Cas9-51-BE	51	BE	20
Exp1	P08	pSSLAlpha9	dsODN-Cas9-51-BE	51	BE	30
Exp1	P09	pSSLAlpha9	dsODN-Cas9-51-BE	51	BE	10
Exp1	P10	pSSLAlpha9	dsODN-Cas9-51-BE	51	BE	20
Exp1	P11	pSSLAlpha9	dsODN-Cas9-51-BE	51	BE	30
Exp1	P12	pSSLAlpha10	dsODN-Cas9-51-OV	51	OV	20
Exp1	P13	pSSLAlpha10	dsODN-Cas9-51-OV	51	OV	30
Exp1	P14	pSSLAlpha10	dsODN-Cas9-51-OV	51	OV	20
Exp1	P15	pSSLAlpha10	dsODN-Cas9-51-OV	51	OV	30
Exp2	P01	pSSLAlpha9	dsODN-Cas9-75-OV	75	OV	10
Exp2	P02	pSSLAlpha9	dsODN-Cas9-75-OV	75	OV	20
Exp2	P03	pSSLAlpha9	dsODN-Cas9-75-OV	75	OV	30
Exp2	P04	pSSLAlpha9	dsODN-Cas9-75-OV	75	OV	10
Exp2	P05	pSSLAlpha9	dsODN-Cas9-75-OV	75	OV	20
Exp2	P06	pSSLAlpha9	dsODN-Cas9-75-OV	75	OV	30
Exp2	P07	pSSLAlpha9	dsODN-Cas9-51-OV	51	OV	10
Exp2	P08	pSSLAlpha9	dsODN-Cas9-51-OV	51	OV	20
Exp2	P09	pSSLAlpha9	dsODN-Cas9-51-OV	51	OV	30
Exp2	P10	pSSLAlpha9	dsODN-Cas9-51-OV	51	OV	10
Exp2	P11	pSSLAlpha9	dsODN-Cas9-51-OV	51	OV	20
Exp2	P12	pSSLAlpha9	dsODN-Cas9-51-OV	51	OV	30
Exp2	P13	pSSLCpf1-3Alpha-1	NA	NA	NA	NA
Exp2	P14	pSSLCpf1-3Alpha-1	NA	NA	NA	NA
Exp3	P01	pSSLAlpha9	dsODN-Cas9-51-OV	51	OV	10
Exp3	P02	pSSLAlpha9	dsODN-Cas9-51-OV	51	OV	20
Exp3	P03	pSSLAlpha9	dsODN-Cas9-51-OV	51	OV	30
Exp3	P04	pSSLAlpha9	dsODN-Cas9-75-OV	75	OV	10

Exp3	P05	pSSLAlpha9	dsODN-Cas9-75-OV	75	OV	20
Exp3	P06	pSSLAlpha9	dsODN-Cas9-75-OV	75	OV	30
Exp3	P07	pSSLAlpha9	dsODN-Cas9-51-BE	51	BE	10
Exp3	P08	pSSLAlpha9	dsODN-Cas9-51-BE	51	BE	20
Exp3	P09	pSSLAlpha9	dsODN-Cas9-51-BE	51	BE	30
Exp3	P10	pSSLAlpha9	dsODN-Cas9-75-BE	75	BE	10
Exp3	P11	pSSLAlpha9	dsODN-Cas9-75-BE	75	BE	20
Exp3	P12	pSSLAlpha9	dsODN-Cas9-75-BE	75	BE	30
Exp3	P13	pSSLCpf1-3Alpha-1	dsODN-Cpf1-51-OV	51	OV	10
Exp3	P14	pSSLCpf1-3Alpha-1	dsODN-Cpf1-51-OV	51	OV	20
Exp3	P15	pSSLCpf1-3Alpha-1	dsODN-Cpf1-51-OV	51	OV	30
Exp3	P16	pSSLCpf1-3Alpha-1	dsODN-Cpf1-75-OV	75	OV	10
Exp3	P17	pSSLCpf1-3Alpha-1	dsODN-Cpf1-75-OV	75	OV	20
Exp3	P18	pSSLCpf1-3Alpha-1	dsODN-Cpf1-75-OV	75	OV	30
Exp4	P01	pSSLAlpha9	dsODN-Cas9-51-OV	51	OV	50
Exp4	P02	pSSLAlpha9	dsODN-Cas9-51-OV	51	OV	100
Exp4	P03	pSSLAlpha9	dsODN-Cas9-51-OV	51	OV	200
Exp4	P04	pSSLAlpha9	dsODN-Cas9-51-BE	51	BE	50
Exp4	P05	pSSLAlpha9	dsODN-Cas9-51-BE	51	BE	100
Exp4	P06	pSSLAlpha9	dsODN-Cas9-51-BE	51	BE	200
Exp4	P07	pSSLAlpha9	dsODN-Cas9-51-OV	51	OV	300
Exp4	P08	pSSLAlpha9	dsODN-Cas9-51-BE	51	BE	300
Exp4	P09	pSSLAlpha10	dsODN-Cas9-51-OV	51	OV	100
Exp4	P10	pSSLAlpha10	dsODN-Cas9-51-OV	51	OV	200
Exp4	P11	pSSLAlpha10	dsODN-Cas9-51-BE	51	BE	100
Exp4	P12	pSSLAlpha10	dsODN-Cas9-51-BE	51	BE	200
